# Wnt-Mediated Repression via Bipartite DNA Recognition by TCF in the *Drosophila* Hematopoietic System

**DOI:** 10.1371/journal.pgen.1004509

**Published:** 2014-08-21

**Authors:** Chen U. Zhang, Timothy A. Blauwkamp, Peter E. Burby, Ken M. Cadigan

**Affiliations:** Department of Molecular, Cellular and Developmental Biology, University of Michigan, Ann Arbor, Michigan, United States of America; New York University, United States of America

## Abstract

The Wnt/β-catenin signaling pathway plays many important roles in animal development, tissue homeostasis and human disease. Transcription factors of the TCF family mediate many Wnt transcriptional responses, promoting signal-dependent activation or repression of target gene expression. The mechanism of this specificity is poorly understood. Previously, we demonstrated that for activated targets in *Drosophila*, TCF/Pangolin (the fly TCF) recognizes regulatory DNA through two DNA binding domains, with the High Mobility Group (HMG) domain binding HMG sites and the adjacent C-clamp domain binding Helper sites. Here, we report that TCF/Pangolin utilizes a similar bipartite mechanism to recognize and regulate several Wnt-repressed targets, but through HMG and Helper sites whose sequences are distinct from those found in activated targets. The type of HMG and Helper sites is sufficient to direct activation or repression of Wnt regulated cis-regulatory modules, and protease digestion studies suggest that TCF/Pangolin adopts distinct conformations when bound to either HMG-Helper site pair. This repressive mechanism occurs in the fly lymph gland, the larval hematopoietic organ, where Wnt/β-catenin signaling controls prohemocytic differentiation. Our study provides a paradigm for direct repression of target gene expression by Wnt/β-catenin signaling and allosteric regulation of a transcription factor by DNA.

## Author Summary

During development and in adult tissues, cells communicate with each other through biochemical cascades known as signaling pathways. In this report, we study the Wnt signaling pathway, using the fruit fly *Drosophila* as a model system. This pathway is known to activate gene expression in cells receiving the Wnt signal, working through a transcription factor known as TCF. But sometimes Wnt signaling also instructs TCF to repress target gene expression. What determines whether TCF will positively or negatively regulate Wnt targets? We demonstrate that activated and repressed targets have distinct DNA sequences that dock TCF on their regulatory DNA. The type of site determines the output, i.e., activation or repression. We find that TCF adopts different conformations when bound to either DNA sequence, which most likely influences its regulatory activity. In addition, we demonstrate that Wnt-dependent repression occurs robustly in the fly larval lymph gland, the tissue responsible for generating macrophage-like cells known as hemocytes.

## Introduction

It is a common theme in gene regulation that the same transcription factor (TF) can directly activate or repress target gene expression, increasing the transcriptional complexity these TFs can achieve [1], [2]. There are several mechanisms by which TFs exhibit this dual regulation. These include TFs interfering with the binding of other TFs to DNA or co-activators [3]–[5] or signal-dependent changes of co-regulators bound to the TF [6]–[8]. In many cases, specific differences in the nucleotide sequence of the cis-regulatory modules (CRMs) targeted by these TFs influence the transcriptional outcome.

The sequence specificity that determines the activation/repression choice of TFs can occur in the TF binding sites themselves, or the surrounding sequences. Several TFs that appear to be intrinsic transcriptional activators can also repress transcription when bound to CRMs in conjunction with other TFs [9]–[11]. In the case of the *Drosophila* NF-κB family member Dorsal, mutation of TF sites flanking Dorsal binding sites converts CRM reporters that are repressed by Dorsal into ones that are activated [12], [13]. For other CRMs regulated by nuclear receptors [14], [15], P53 [16], the POU TF Pit1 [17] and some Smads [18], [19], it is the type of the TF binding site itself that determines output. For the latter cases, it has been proposed that the DNA binding site allosterically regulates the TF, leading to differential recruitment of co-regulators [17], [20].

Dual regulation of transcription has also been seen in Wnt/β-cat (hereafter called Wnt) signaling, an important cell-cell communication pathway that plays various roles throughout animal development, stem cell biology and disease [21]–[23]. Wnt-induced nuclear accumulation of β-catenin (β-cat) is a key feature of this pathway. Once in the nucleus, β-cat is recruited to CRMs hereafter referred to as Wnt-dependent CRMs (W-CRMs), where it facilitates regulation of Wnt transcriptional targets [24], [25].

The best-characterized TFs that recruit β-cat to W-CRMs are members of the T-cell factor (TCF) family [26]. Studies with synthetic W-CRMs containing multiple copies of high affinity TCF binding sites and mutagenesis studies of binding sites in many endogenous W-CRMs support the view that TCF/β-cat complexes are powerful transcriptional activators [26]–[28]. In many cases, TCFs also mediate default repression by binding to W-CRMs in the absence of signaling [23], [28]. This regulation is commonly referred to as the TCF “transcriptional switch” [1], [28]. While vertebrate TCFs have become more specialized for either default repression or β-cat-dependent activation, invertebrate TCFs such as *Drosophila* TCF/Pangolin (TCF/Pan) mediate both sides of the transcriptional switch [26], [28].

All TCFs contain a sequence-specific DNA binding domain called the HMG domain, whose high affinity consensus is SSTTTGWW, (S = C/G, W = A/T) [29]–[31]. Invertebrate TCFs and some vertebrate TCF isoforms contain a second DNA binding domain, C-terminal to the HMG domain, known as the C-clamp [26], [32]. C-clamps recognize GC-rich motifs called Helper sites, and this interaction is essential for the activation of many W-CRMs [33], [34]. These data support a model where C-clamp containing TCFs recognize W-CRMs in a bipartite manner, via HMG domain-HMG site and C-clamp-Helper site interactions [26].

While TCF/β-cat complexes are commonly associated with transcriptional activation, there are a few cases where they appear to directly repress target gene expression [35]–[38]. The HMG sites in these repressed W-CRMs are very similar to those found in activated targets. In one case, TCF/β-cat may achieve repression by interfering with the binding of another activating TF [35]. For another target, TCF/β-cat may form a complex with the transcriptional repressor Brinker, and HMG and Brinker binding sites are both required for the repression [38].

In contrast to the aforementioned examples, we previously showed that TCF/Pan mediated Wnt-dependent repression of a W-CRM from the *Ugt36Bc* locus through HMG sites with a consensus that is distinct (WGAWAW) from classic ones [39]. In addition to mediating Wnt-induced repression, TCF/Pan is required for basal expression of *Ugt36Bc* in the absence of signaling [39]. This suggests a “reverse transcriptional switch” occurs at *Ugt36Bc* compared to the switch seen in activated targets. Instead of TCF/Pan default repression and Wnt-dependent activation, the reverse switch consists of TCF/Pan basal activation and Wnt-dependent repression.

In this report, we have explored the mechanism of this reverse switch/direct repression mechanism by TCF/Pan and Wnt signaling in more detail. We identified another repressed W-CRM from the *Tiggrin* (*Tig*) gene, which contains functional WGAWAW sites bound by TCF/Pan. Regulation of the *Ugt36Bc* and *Tig* W-CRMs by TCF/Pan requires the C-clamp, which binds to Helper-like (r-Helper) sites adjacent to the WGAWAW sites. Swapping these sites in the *Tig* W-CRM to classic HMG and Helper sites converts the W-CRM into one that is activated by Wnt signaling. Conversely, an activated W-CRM from the *naked cuticle* (*nkd*) locus was converted to a repressed W-CRM by replacing its classic HMG-Helper pairs with pairs from the *Tig* W-CRM. Partial protease digestion indicates that TCF/Pan adopts a different conformation when bound to classic or repressive sites, supporting allosteric regulation of TCF/Pan by its binding sites. In addition, we have extended this work from cell culture to the fly, showing that WGAWAW and r-Helper sites mediate basal activation and Wnt-induced repression in the larval lymph gland (LG). Wnt signaling is known to play an important role in regulating hematopoiesis in the LG [40]. Thus, our work provides insight into how TCF/Pan can activate and repress Wnt transcriptional targets, and extends the TCF reverse transcriptional switch mechanism to a physiologically relevant context.

## Results

### Regulation of Wnt-repressed targets requires the C-clamp of TCF/Pan

*Ugt36Bc* was originally identified as a candidate for repression by Wnt signaling from a microarray screen performed in Kc167 (Kc) cells [39], a *Drosophila* cell line likely of hemocytic origin [41]. Several other repressed targets were also identified in this screen, including *Tig* [39], which encodes an extracellular matrix protein that serves as a PS2 integrin ligand [42], [43]. *Tig* expression was repressed by DisArmed, a mutated version of Armadillo (Arm, the fly β-catenin) which is defective in gene activation but is still competent for repression [39]. While these results are consistent with *Tig* being directly repressed by Wnt signaling, the cis-regulatory information responsible for Wnt regulation of *Tig* expression had not been identified.

**Figure 1:**
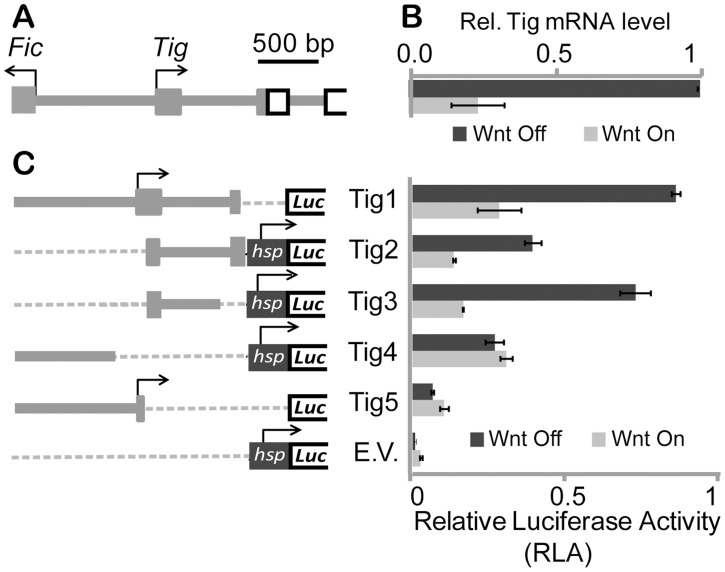
Characterization of *Tig* cis-regulatory information in Kc cells. (**A**) Cartoon depicting the intergenic region between the *Tig* and *Fic* loci. Bent arrows represent the TSSs of each gene, grey boxes the 5′ UTRs, and white rectangles the *Tig* ORF. (**B**) *Tig* transcript levels in Kc cells are repressed when Wnt signaling is activated via Axin RNAi as previously described [39]. (**C**) The Tig reporters assayed are depicted on the left. The *hsp70* (*hsp*) promoter is not drawn to scale. Regulation of the luciferase reporters by Wnt signaling (using Axin RNAi) in Kc cells is shown in the graph on the right. See Materials and Methods for details of the transfection conditions.

The *Tig* locus is compact, with a small (∼1 kb) intergenic region and six introns, only the first of which is larger than 500 bp (Figure 1A). The intergenic region possibly also contains elements driving the expression of the adjoining gene, *Fic domain-containing protein* (*Fic*), a gene involved in fly vision [44]. *Fic* was expressed in Kc cells, but was not regulated by Wnt signaling (Figure S1B). A 1.8 kb fragment containing the intergenic region between *Fic* and *Tig*, as well as the first exon and intron and part of the second exon of *Tig* was cloned upstream of a luciferase gene reporter (Figure 1C). This reporter (Tig1) was repressed 2–5 fold by Axin RNAi in Kc cells, similar to the fold regulation of endogenous *Tig* mRNA (Figure 1B and 1C). Expression of a stabilized form of Arm (Arm*) [45] also repressed the Tig1 reporter to a similar degree (Figure S1C). These results suggest that Tig1 contains most of the regulatory information required for Wnt regulation of the *Tig* gene.

To better understand which regions were responsible for basal expression and Wnt-dependent repression of *Tig*, smaller fragments of the regulatory sequences in Tig1 were analyzed. In some cases (Tig2–Tig4), sequences were cloned upstream of the *hsp70* core promoter, which is unregulated by Wnt signaling [33], [39], [45], while the Tig5 reporter used the endogenous *Tig* promoter. These reporters (Tig2–Tig5) all had basal expression higher than the *hsp70* promoter control (Figure 1C). Much of the repressive activity appeared to be contained in a 578 bp fragment containing part of the first exon and most of the first intron (Tig3). However Tig1 was used for further functional experiments, to retain the endogenous promoter and additional cis-regulatory information of the *Tig* locus.

**Figure 2:**
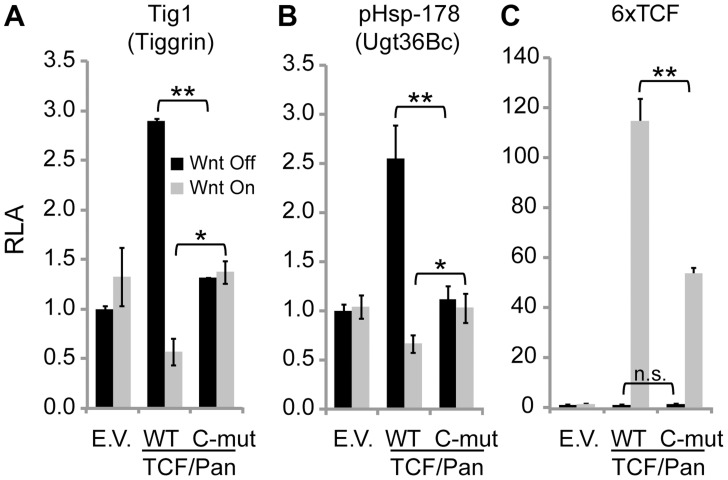
The C-clamp domain of TCF/Pan is required for Wnt-mediated repression of *Tig* and *Ugt36Bc* W-CRMs. TCF rescue assays in Kc cells were performed as previously described [33]. Endogenous TCF was depleted with dsRNA corresponding to the *TCF/Pan* 3′UTR for four days before co-transfection of W-CRM reporters with empty expression vector (E.V.) or ones expressing wild-type (WT) TCF/Pan or TCF/Pan containing five amino acid substitutions in the C-clamp (C-mut). Wnt signaling was activated by the over-expression of Arm*. (**A**) The Tig1 reporter is not regulated in TCF/Pan depleted cells. Transfection of WT TCF/Pan rescues basal activation and Wnt-mediated repression, but the C-clamp mutant variant does not. (**B**) The *Ugt* W-CRM reporter pHsp-178 [39] behaved similarly to as Tig1. For both reporters, WT TCF/Pan repressed expression to significantly lower levels than the C-clamp mutant (compare the fourth and sixth bars). (**C**) Activation of a synthetic reporter containing six classic HMG binding sites (6×TCF) was rescued by wild-type TCF, while the C-clamp mutant rescued activation about half as well. In each experiment, luciferase activity in the absence of Wnt signaling without TCF expression was normalized to 1.0 for each reporter. *P<0.05. **P<0.01. n.s., not significant (Student's T-test).

TCF/Pan has previously been shown to activate *Ugt36Bc* and *Tig* in the absence of signaling, and to be required for Wnt-mediated repression [39]. To determine whether the C-clamp of TCF/Pan was required for these activities, RNAi rescue experiments were performed. Endogenous TCF/Pan was depleted from Kc cells using dsRNA corresponding to the 3′ UTR of *TCF/Pan*. Cells were then transfected with *Ugt36Bc* or *Tig* reporters, as well as expression plasmids for TCF/Pan, either wild-type control or a C-clamp mutant where five amino acids have been altered [33]. Wnt signaling was activated using Arm*. In control TCF/Pan depleted cells (transfected with empty vector), the *Tig* and *Ugt36Bc* reporters were not regulated by Arm* (Figure 2A and 2B). Wild-type TCF/Pan elevated basal expression and enabled significant repression by Arm*. In contrast, the C-clamp mutant neither activated nor repressed the reporters (Figure 2A and 2B). These data suggest that the C-clamp is required for TCF/Pan-dependent basal activity and Wnt-mediated repression of both reporters.

To ensure that the C-clamp mutant TCF/Pan was functional, a synthetic reporter containing multimerized HMG sites and lacking Helper sites (6×TCF) was also examined (Figure 2C). As previously reported [33], the C-clamp mutant was able to rescue 6×TCF activation by Wnt signaling, albeit not completely under the conditions used (Figure 2C). Nonetheless, these data support an important role for the C-clamp in TCF/Pan regulation of the *Ugt36Bc* and *Tig*.

### *Tig* and *Ugt36Bc* W-CRMs both contain distinct HMG and Helper sites

**Figure 3:**
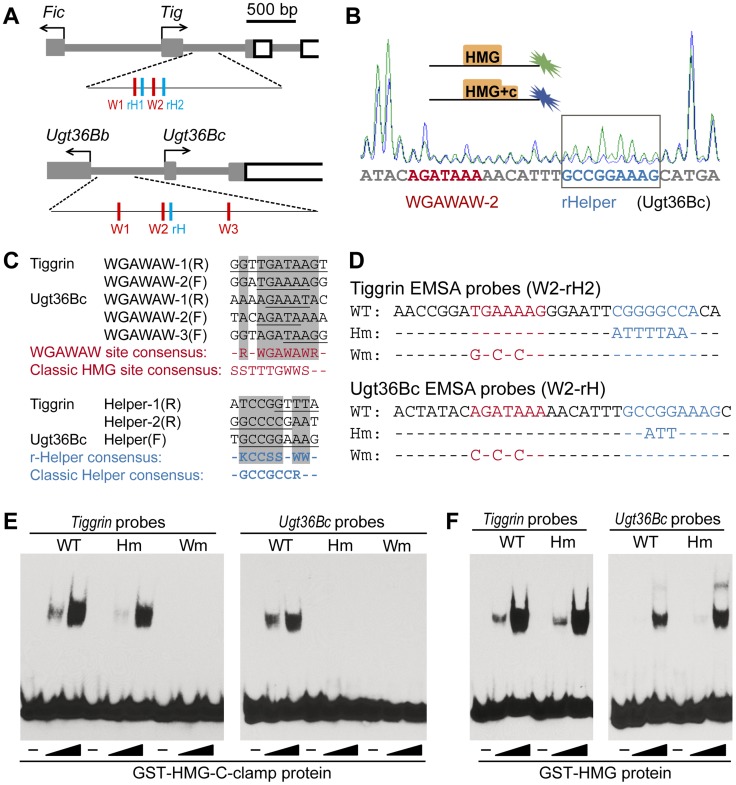
TCF recognizes repressed W-CRMs through a bipartite mechanism. (**A**) A cartoon showing the *Tig* and *Ugt36Bc* loci, along with the regions that were footprinted indicating the location of the WGAWAW sites (red) and r-Helper sites (blue). (**B**) Example of a footprinting chromatograph showing the C-clamp-specific protection of the r-Helper in the *Ugt36Bc* W-CRM. The boxed region where the green peaks are higher than the blue indicates sequences protected by GST-HMG-C-clamp and not by GST-HMG. (**C**) Alignment of the WGAWAW and r-Helper sites identified by footprinting from the *Tig* and *Ugt36Bc* W-CRMs. The WGAWAW sites were identified by comparing footprints of GST-HMG and GST, while r-Helper sites were footprinted by GST-HMG-C-clamp and not GST-HMG. In the alignments, the footprinted sequences are underlined. The consensuses for each motif are shown, along with the classic HMG and Helper site consensuses. (**D**) Sequences of the probes used for EMSA, derived from two endogenous WGAWAW, r-Helper pairs. Mutations in the r-Helper and WGAWAW motifs are indicated. (**E**) EMSA data showing that both WGAWAW sites and r-Helper sites were required for maximal binding with GST-HMG-C-clamp protein. The reduction of binding with the Tig Hm probe was slight but reproducible. (**F**) EMSA showing that r-Helper sites were not required for binding by GST-HMG protein. All footprinting and EMSA experiments were performed at least three times with similar results.

A search through the Tig1 sequences using the open access program Target Explorer [46] failed to find classic HMG sites (SSTTTGWWS) [29], [31] or the Helper sites characterized in activated fly W-CRMs (GCCGCCR) [33]. However, the first intron of *Tig* contained several sequences that were similar to sites in the *Ugt36Bc* W-CRM that were footprinted by the HMG domain of TCF/Pan [39]. Therefore, similar footprinting of a 300 bp region of the *Tig* intron containing these putative sites (Figure 3A) was performed, comparing the footprint of GST and GST-HMG domain recombinant proteins (see Material and Methods for details). Several regions of this *Tig* regulatory DNA were protected by the HMG domain (Figure S2A), two of which are similar to the three WGAWAW sites previously found in the *Ugt36Bc* W-CRM [39]. Together, the five *Tig* and *Ugt36Bc* motifs defined a consensus of RNWGAWAW (Figure 3C). In addition, the regions of the *Ugt36Bc* and *Tig* loci containing the WGAWAW sites were footprinted with GST-HMG and GST-HMG-C-clamp, to identify C-clamp bound sequences. Three additional regions were protected only in the presence of the C-clamp (Figure 3B, S2A and S3). Alignment of these regions revealed a consensus of KCCSSNWW (K = G/T; Figure 3C), which was distinct from the classic Helper sites found in activated W-CRMs. These motifs are hereafter referred to as repressive-Helper (r-Helper) sites and the HMG bound sequences as WGAWAW sites.

The r-Helper sites in the *Ugt36Bc* and *Tig* W-CRMs are adjacent to the WGAWAW sites (Figure 3A), similar to the HMG-Helper clustering in activated W-CRMs [33], [34]. To test whether these motifs act together to form a high affinity binding site for TCF/Pan, labeled probes containing a WGAWAW-r-Helper pair from *Tig* and *Ugt36Bc* were synthesized (Figure 3D) and analyzed for binding to recombinant GST-TCF/Pan fusion proteins using EMSA (Electrophoretic Mobility Shift Assay). Both probes were bound by GST-HMG-C-clamp, and mutation of the WGAWAW site abolished binding (Figure 3E). Mutation of the r-Helper site abolished binding in the case of the *Ugt36Bc* probe, and resulted in a small but reproducible reduction in binding of the *Tig* probe (Figure 3E). This difference was also seen with the footprinting data, where GST-HMG-C-clamp protection of the *Ugt36Bc* r-Helper site (Figure 3B) was more pronounced than the r-Helper sites in the *Tig* W-CRM (Figure S3). Consistent with being C-clamp binding sites, the r-Helper motifs were not required for binding by GST-HMG protein (Figure 3F). Taken together, these data support a model in which TCF/Pan binds to the *Ugt36Bc* and *Tig* W-CRMs through bipartite binding of HMG domain to WGAWAW sites and C-clamp binding to r-Helper sites.

**Figure 4:**
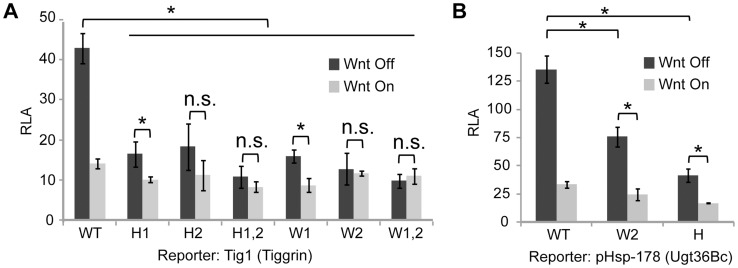
r-Helper and WGAWAW sites are required for Wnt-regulation of *Tig* and *Ugt36Bc* W-CRM reporters. (**A, B**) Mutations in r-Helper sites (H) or WGAWAW sites (W) greatly decrease the basal activity and repression of the *Tig* and *Ugt36Bc* W-CRM reporters in Kc cells by Axin RNAi (A, B) or Arm* expression (Figure S1C and S1D). *p<0.05; n.s., not significant (Student's T-test).

To determine whether the WGAWAW and r-Helper sites in the *Tig* W-CRM were functional, site-directed mutagenesis of the Tig1 reporter was performed. Altering either WGAWAW or r-Helper sites resulted in a strong reduction of basal expression and Wnt-dependent repression (Figure 4A). These data were similar to those obtained when the WGAWAW sites in the pHsp-178 *Ugt36Bc* reporter were altered [39]. When the r-Helper site in pHsp-178 was mutated, a similar defect was observed as when the adjacent WGAWAW site was destroyed (Figure 4B). These data demonstrate that the distinct bipartite TCF/Pan binding sites found in the *Tig* and *Ugt36Bc* W-CRMs are necessary for basal expression of the reporters. In the absence of these motifs, Wnt signaling causes little reduction in expression of these reporters, either due to loss of basal expression and/or loss of active repression by the pathway.

In addition to the two WGAWAW sites in the *Tig* intronic W-CRM, five additional sequences were footprinted by the HMG domain, most of which were enriched with a TG-rich motif (Figure S2A). All five motifs were mutated, but the expression of these mutant reporters were not affected in a significant manner (Figure S2B). While it is possible that these motifs are functionally redundant, they were not analyzed further in this study.

### The type of HMG and Helper sites determines transcriptional output of TCF/Pan through allosteric regulation

**Figure 5:**
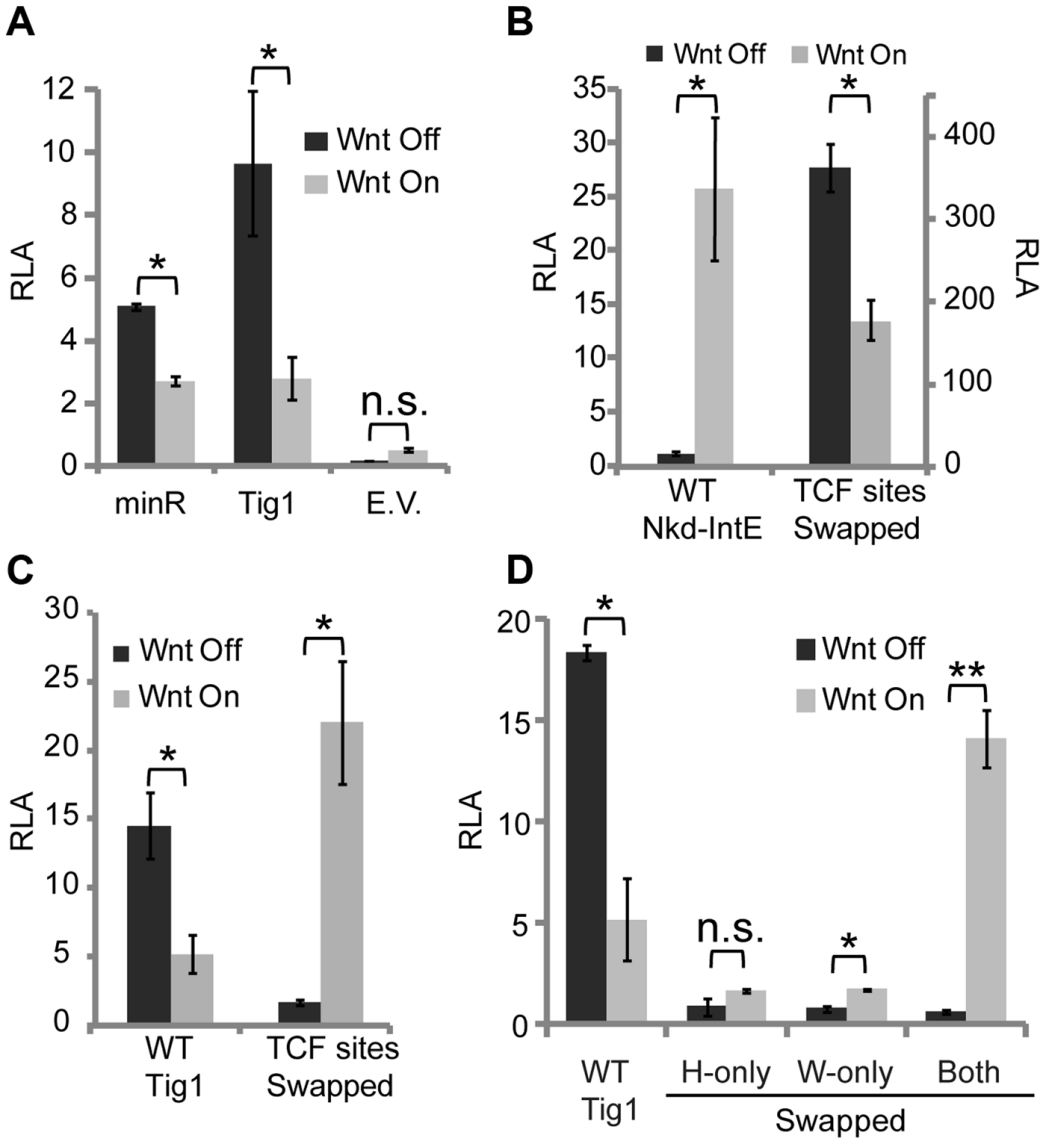
Swapping HMG and C-clamp binding sites switches the transcriptional output of W-CRMs. Kc cells were transfected with the indicated reporters with or without Axin RNAi, as described in Figure 1 and the Materials and Methods. Sequences of the reporters used are listed in Figure S3. (**A**) A minR reporter containing two repeats of a 40 bp region of the *Tig* intron (each repeat contains two WGAWAW and two r-Helper sites) cloned upstream of the *hsp70* core promoter is sufficient for driving basal expression and mediating Wnt repression. Tig1 and the *hsp70* core promoter (E.V.) were used as positive and negative controls, respectively. (**B**) The *nkd*-IntE W-CRM reporter, which is activated by Wnt signaling, is converted to a repressed W-CRM when its three functional HMG sites and two Helper sites were replaced by five WGAWAW and r-Helper pairs (see Figure S3 for sequence changes). (**C**) The Tig1 W-CRM reporter is activated by Wnt signaling when two WGAWAW sites and two r-Helper sites were converted into classic HMG-Helper pairs. (**D**) The switch of the Tig1 W-CRM to an activated W-CRM requires swapping both WGAWAW and r-Helper sites. When one motif is swapped without the other, low basal activity and little activation was observed. *p<0.05; **p<0.01; n.s.: not significant (Student's T-test).

Since WGAWAW and r-Helper sites contribute to both basal activation and Wnt-mediated repression of *Tig* and *Ugt36Bc* W-CRMs (Figure 4) [39], these bipartite TCF binding sites could be sufficient for this regulation. To test this, a synthetic reporter containing two repeats of a small stretch (40 bp) from the *Tig* W-CRM (each repeat contains two pairs of WGAWAW and r-Helper sites) was constructed (Figure S4A). This reporter, termed “minR” for “minimal repressed W-CRM”, was repressed about two-fold by Axin RNAi or Arm* expression in Kc cells (Figure 5A; Figure S5A). Like the *Tig* and *Ugt36Bc* W-CRMs, the basal expression of the minR reporter is dependent on the WGAWAW and r-Helper sites (Figure S5B). These results demonstrate that these bipartite TCF sites are necessary and sufficient for the “reverse TCF/Pan transcriptional switch” that regulates targets repressed by Wnt signaling.

The behavior of minR is the qualitative opposite of classic HMG-Helper site pairs, which are highly activated by Wnt signaling [33]. This suggests that the TCF/Pan sites themselves dictate whether a W-CRM is activated or repressed by the Wnt pathway. To test this, the HMG-Helper sites in the *nkd*-IntE W-CRM, which is activated by Wnt signaling in Kc cells and flies [33], [47], were replaced by WGAWAW-r-Helper sites (see Figure S4B for base pair changes). The basal activity of this “TCF sites swapped” *nkd*-IntE was significantly higher than either the original *nkd*-IntE or minR, suggesting a synergistic effect between the repressive TCF sites and the remaining sequences of *nkd*-IntE (Figure 5B). Strikingly, this W-CRM was repressed upon activation of Wnt signaling (Figure 5B).

To determine whether the *Tig1* W-CRM could be converted into an activated W-CRM, the functional WGAWAW and r-Helper sites identified in Figure 4 were converted into classic HMG and Helper sites (Figure S4C). This swapped Tig1 reporter was robustly activated by Wnt signaling (Figure 5C). To assess the individual contribution of each type of binding site to the switch in transcriptional output, r-Helper site only (H-only) and WGAWAW site only (W-only) swaps were constructed in the Tig1 reporter (Figure S4C). These “partial swap” W-CRMs lost the high basal expression of Tig1, and lacked the high activation seen when both motifs are swapped (Figure 5D). Taken together, these data argue that both the HMG domain and C-clamp binding domains are instructive in determining whether a W-CRM is activated or repressed by Wnt signaling.

**Figure 6:**
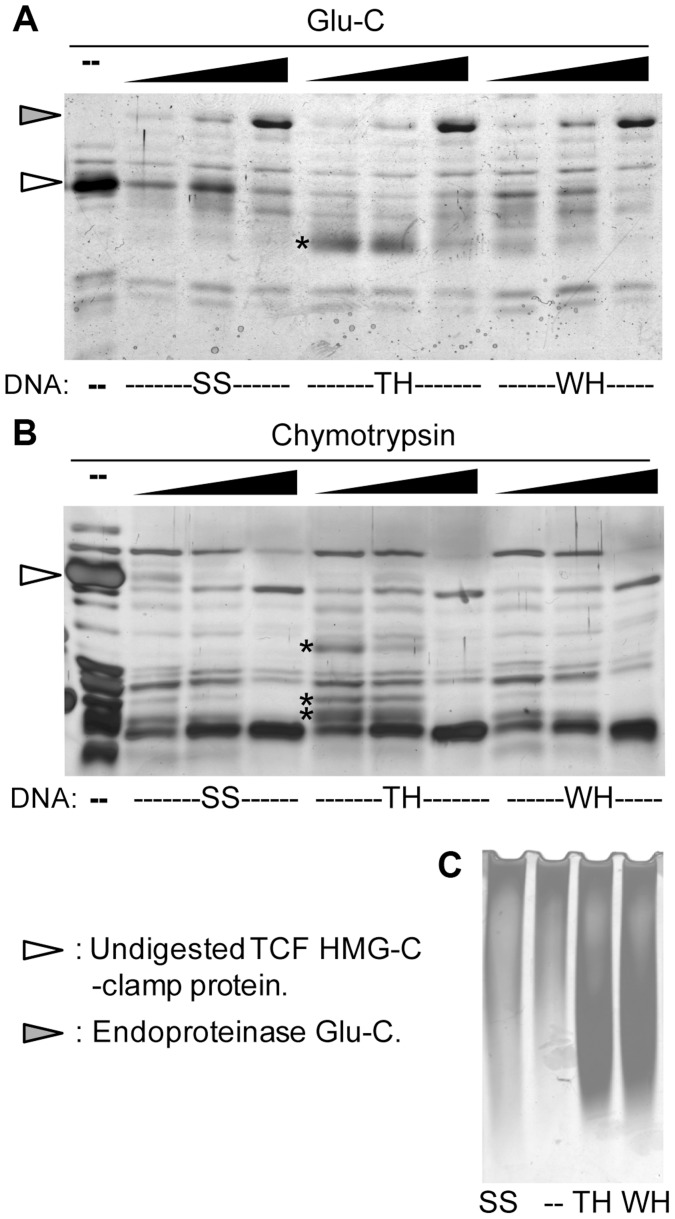
The HMG and C-clamp domains adopt different conformations when bound to distinct binding sites. (**A, B**) Recombinant GST-HMG-C-clamp protein was incubated with 20 fold molar excess of control oligonucleotide (SS), a classic HMG and Helper site pair (TH) and a WGAWAW and r-Helper site pair (WH) (see Table S2 for sequences of oligonucleotides). After 20 min to allow binding, the preps were subjected to partial proteolytic digestion with increasing amounts of Glu-C (A) or chymotrypsin (B) and analyzed by SDS-PAGE followed by silver staining. Proteolytic fragments enriched with TH and not WH are indicated with asterisks. (**C**) Silver stained native gel of GST-HMG-C-clamp and different oligonucleotides at the same concentrations used in the proteolytic digestions, demonstrating that a similar amount of protein is bound to TH and WH, while SS has no detectable binding. Each experiment was performed at least three independent times with similar results.

Our findings that the transcriptional output can be reprogrammed by altering the TCF binding sites suggests that DNA is allosterically regulating TCF/Pan. To test this, recombinant HMG-C-clamp protein was incubated with excess oligonucleotides containing activating or repressed TCF sites followed by partial digestion with two proteases, chymotrypsin or endoproteinase Glu-C. The digested product was then separated on SDS-PAGE gels. The digestion patterns between HMG-C-clamp bound with a classic HMG-Helper site pair (TH) and WGAWAW-r-Helper pair (WH) were distinct, with several proteolytic fragments observed with TH that were not detectable with WH (Figure 6A and 6B). Analyzing HMG-C-clamp mobility on a native gel indicates that the majority of the protein was complexed with either the TH [33] or WH probe (compare the shift with a control SS probe which does not bind TCF in Figure 6C). These data strongly suggest that the conformations of the HMG and/or C-clamp domains are distinct when bound to activating or repressing TCF sites.

The HMG domain of LEF1 (a vertebrate TCF) is known to induce a sharp bend in DNA when bound to a classic HMG site [48]. Therefore, the possibility exists that differences in DNA bending could contribute to the transcriptional specificity of activated and repressed W-CRMs. To address this, probes where the position of the binding site was altered were tested via EMSA (Figure S6). If protein binding induced a bend in the DNA, mobility will be slowest when the binding site was present in the middle of the probe [49]. Consistent with the LEF1 data, the HMG domain of TCF/Pan exhibited bending when bound to a classic HMG site (Figure S6B). In addition, GST-HMG could bend a WGAWAW site probe, though the bend was slightly less than the classic HMG site (Figure S6B). The presence of a C-clamp in the protein and a Helper site in the probe did not alter the degree of bending (Figure S6C). Likewise the reduction of bending of the WGAWAW site was still observed when paired with an r-Helper site and bound by GST-HMG-C-clamp (Figure S6D). The data demonstrated a small difference in bending between the activated and repressed binding sites, which could contribute to the transcriptional specificity.

### Natural and synthetic WGAWAW, r-Helper containing W-CRMs function in the *Drosophila* hematopoietic system

**Figure 7:**
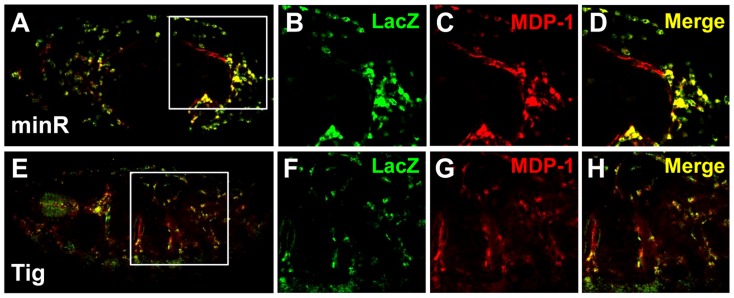
Embryonic expression of the Tig and minR reporters. (**A–D**) Micrographs of a stage 14 embryo containing a minR lacZ reporter immunostained for LacZ (green) and the hemocytic marker MDP-1 (red). Panel A shows the entire embryo while panels B–D are higher magnification insets (white box in A). The majority of lacZ staining is hemocytic. (**E–H**) Stage 16 embryo containing a Tig1 lacZ reporter stained and presented as in panels A–D. There is significant overlap between the reporter expression and hemocytes.

**Figure 8:**
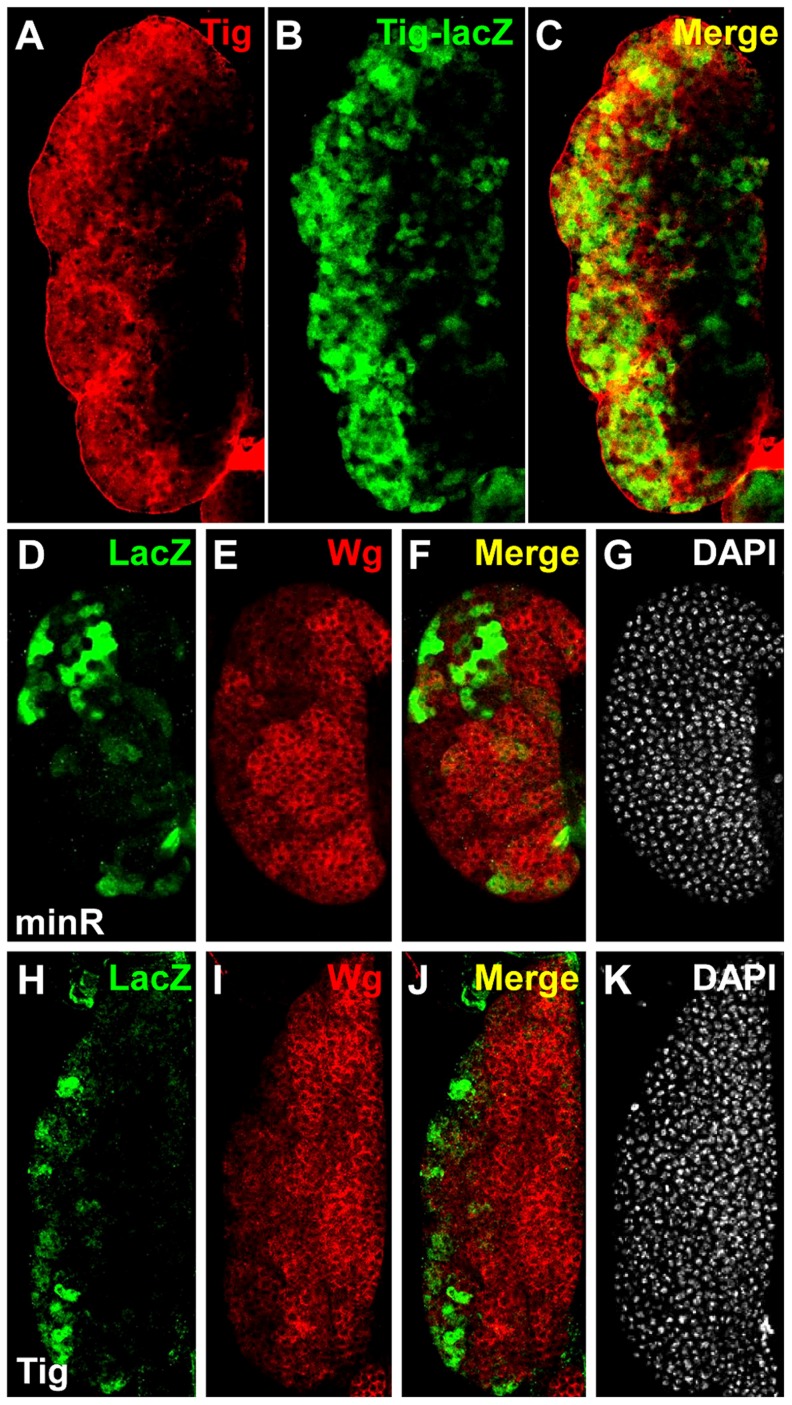
Expression of *Tig* and minR reporters in the larval LG. (**A–C**) Larval LG from older late 3^rd^ instar larvae (∼104–112 AEL) containing the Tig lacZ reporter immunostained for Tig protein (green) and LacZ (red). The red and green signals colocalize to the same cells, with most Tig localized extracellularly and LacZ to the cytosol. (**D–K**) Larval LGs from younger late 3^rd^ instar larva (∼96–104 AEL) containing the minR (D–G) or Tig1 (H–K) lacZ reporters, immunostained for LacZ (green) and Wg (red). DAPI was used as a counterstain (white). The expression patterns of the reporters and Wg are largely exclusive, suggesting that the reporters are repressed by Wnt signaling.

To extend the analysis of Tig1 and minR reporters to the whole organism, these W-CRMs were cloned into P-element Pelican vectors [50], carrying the LacZ reporter gene plus insulators to minimize position effects, either using the endogenous *Tig* promoter (Tig1) or a heterologous one from *hsp70* (minR). Transgenic lines were established and analyzed for LacZ expression in embryos and larva. Both reporters were active in embryonic hemocytes, as indicated by co-localization with MDP-1, a hemocyte marker (Figure 7A–7H) [51]. We also found staining of both reporters in the larval lymph gland (LG), fat body and circulating hemocytes (Figure 8; data not shown). These patterns are similar to that of endogenous Tig in the LG (Figure 8A–8C), as well as embryonic hemocytes and fat body [42]. These results indicate that both reporters can be used to study regulation by Wnt signaling in vivo.

The Tig1 and minR reporters are both expressed at much higher levels in the cortical zone (CZ) of the LG, an irregularly shaped region containing mature hemocytes enriched in the periphery of the LG (Figure 8B, 8D, 8H). This pattern is largely non-overlapping with Wingless (Wg, a fly Wnt), which is enriched in the medullary zone (MZ) containing prohemocytes [40] (Figure 8E and 8I). The Wg pattern is more apparent in younger late 3^rd^ instar larvae, i.e., ∼96–104 after egg laying (∼96–104 AEL; Figure 8D–8K), but the lacZ reporters expressed highest in older late 3^rd^ instar larvae (∼104–112 AEL; Figure 8A–8C). The expression of the reporters did not overlap with Lozenge-Gal4≫UAS-GFP (Lz≫GFP), which marks crystal cells, a hemocyte lineage found in the CZ that often has high Wg expression [40] (Figure S7). While the presence of Wg in the MZ doesn't necessarily imply active Wnt signaling, these results support a model where Wnt signaling represses Tig and minR expression in this portion of the LG.

**Figure 9:**
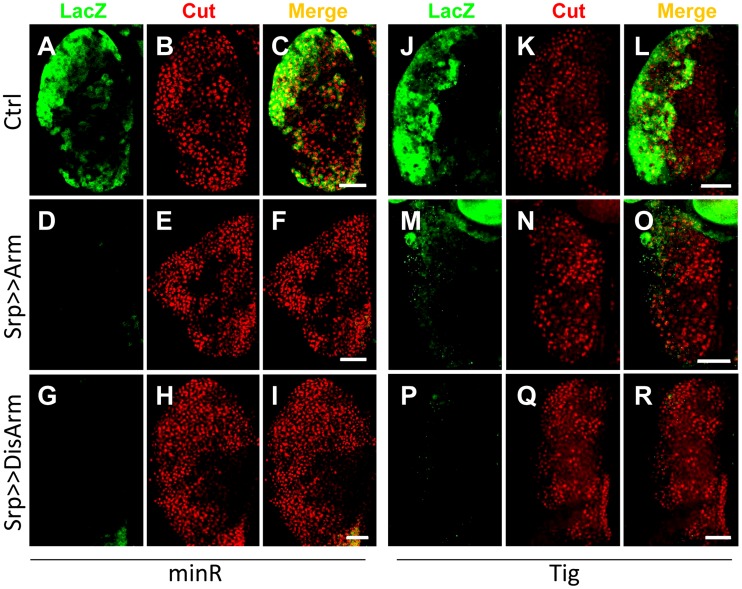
The *Tig* and minR reporters are repressed by Wnt signaling in the larval LG. Micrographs of older late 3^rd^ instar larval LGs from strains containing the minR (**A–I**) or Tig (**J–R**) lacZ reporters, combined with P[UAS-Arm*] (D–F, M–O) or P[UAS-DisArmed] (G–I; P–R) transgenes driven by P[Srp-Gal4]. The green signal denotes LacZ and red is Cut, a marker for the CZ [53]; [Figure S8]. Activation of Wnt signaling by Arm* or DisArmed expression inhibits reporter expression without detectably altering the size of the CZ. Bar = 40 µm.

To test whether the Tig1 and minR reporters were repressed by Wnt signaling in the LG, the Gal4 misexpression system [52] was used to modulate the Wnt pathway. Serpent-Gal4 (Srp-Gal4), which is active throughout the LG [53], was combined with UAS lines expressing Arm* or DisArmed in a background containing either reporter. Expression of either Arm* or DisArmed in the LG repressed the minR (Figure 9A, 9D and 9G) and Tig (Figure 9J, 9M and 9P) reporters with 100% penetrance. Under the conditions employed, no detectable change in expression of Cut, a CZ marker (Figure S8) [53], was observed (Figure 9B, 9E, 9H, 9K, 9N and 9Q), ruling out a gross change in cell fate in the LG being responsible for the loss of reporter expression. With stronger or longer expression of Arm*, we did observe a strong reduction of the CZ cell fate as previously reported (Figure S9) [40]. The results indicate that Wnt signaling can repress the *Tig* and minR reporters in the CZ without detectably altering cell fate. In addition, the finding that DisArmed can mediate this regulation suggests that the transcriptional activation activity of Arm is not required for this regulation.

**Figure 10:**
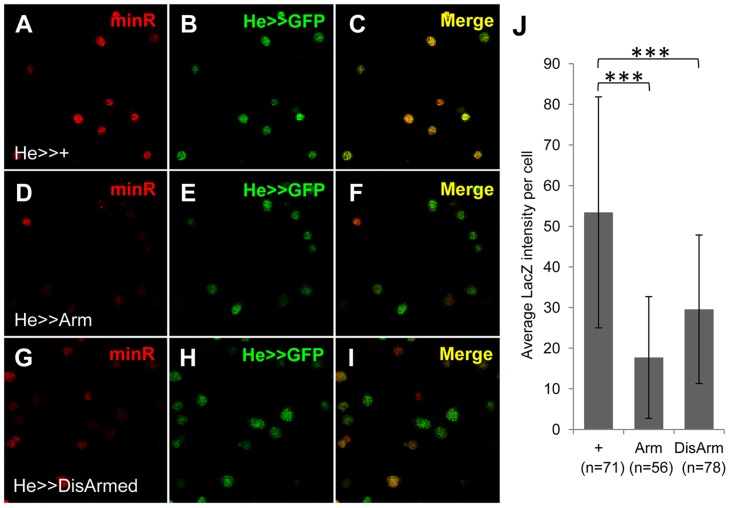
The minR reporter is repressed by Wnt signaling in circulating larval hemocytes. (**A–I**) Micrographs of mid 3^rd^ instar larval (∼88–96 AEL) circulating hemocyte smears from strains containing the minR reporter, P[He-Gal4] and P[UAS-GFP] and either + (A–C), P[UAS-Arm*] (D–F) or P[UAS-DisArmed] (G–I) transgenes. Activation of Wnt signaling by Arm* or DisArmed expression inhibits reporter expression in most of the circulating hemocytes. (**J**) Quantification of the data (see Materials and Methods) using 5 larvae for each genotype and 10–15 hemocytes per larvae. ***p<0.001.

To test whether the Tig1 and minR reporters were repressed by Wnt signaling in embryonic hemocytes, we expressed Arm* or DisArmed under the control of two embryonic hemocyte drivers, Srp-Gal4 or Croquemort-Gal4 (Crq-Gal4). No detectable repression was observed (data not shown). To examine whether the negative results were due to perdurance of LacZ, we assayed circulating hemocytes from mid 3^rd^ instar larvae (∼88–96 AEL). This is prior to release of LG hemocytes, so all circulating hemocytes are of embryonic lineage at this developmental stage [54]. Hemese-Gal4 (He-Gal4) [55], a circulating hemocyte driver, was used to drive the expression of UAS-Arm* or UAS-DisArmed. Expression of either transgene resulted in a significant repression of the minR reporter (Figure 10), demonstrating Wnt repression of this reporter in the embryonic hemocyte lineage.

Our working model is that TCF/Pan activates Tig1 and minR expression in the CZ of the LG, while Wnt signaling represses these reporters in the MZ. To test this, we examined reporter expression when dominant-negative versions of Frizzled and Frizzled2 (Fz^DN^ and Fz2^DN^) [56], [57] were expressed via the MZ driver Dome-Gal4 [58]. We observed a strong expansion of minR in these LGs, but there was also a concomitant expansion of the CZ, indicated by a reduction of Dome≫GFP (Figure S10). This is consistent with a previous report demonstrating that Wnt signaling is required for maintenance of the MZ [40]. Depletion of TCF/Pan in the CZ using RNAi caused the predicted reduction in reporter gene expression, but there was also a reduction in the CZ (Figure S11). In both cases, the change in reporter expression was coupled with a change in cell fate, preventing a definitive demonstration that endogenous TCF/Pan and Wnt signaling regulates the minR and Tig reporters in the LG (see Discussion for further comment).

**Figure 11:**
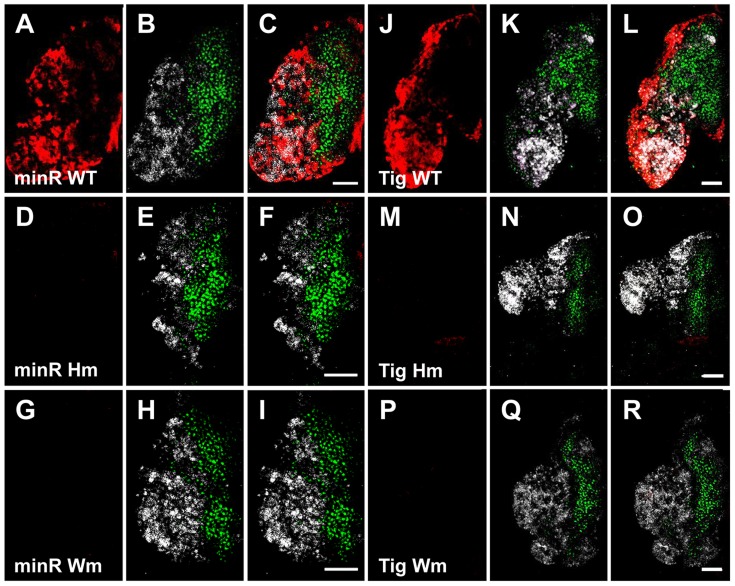
The TCF binding sites are required for expression of the *Tig* and minR reporters in the CZ of larval LG. (**A–R**) Older late 3^rd^ instar larval LGs from minR (A–I) and Tig1 (J–R) reporters with mutations in the r-Helper (Hm) or WGAWAW sites (Wm). Mutation of either motif abolishes LG expression for both reporters, indicated by LacZ signal in red. The Dome≫EBFP (green) and Hml≫dsRed (white) mark the MZ and CZ, respectively. When active, the LacZ signal is found in the CZ. Bar = 50 µm.

**Figure 12:**
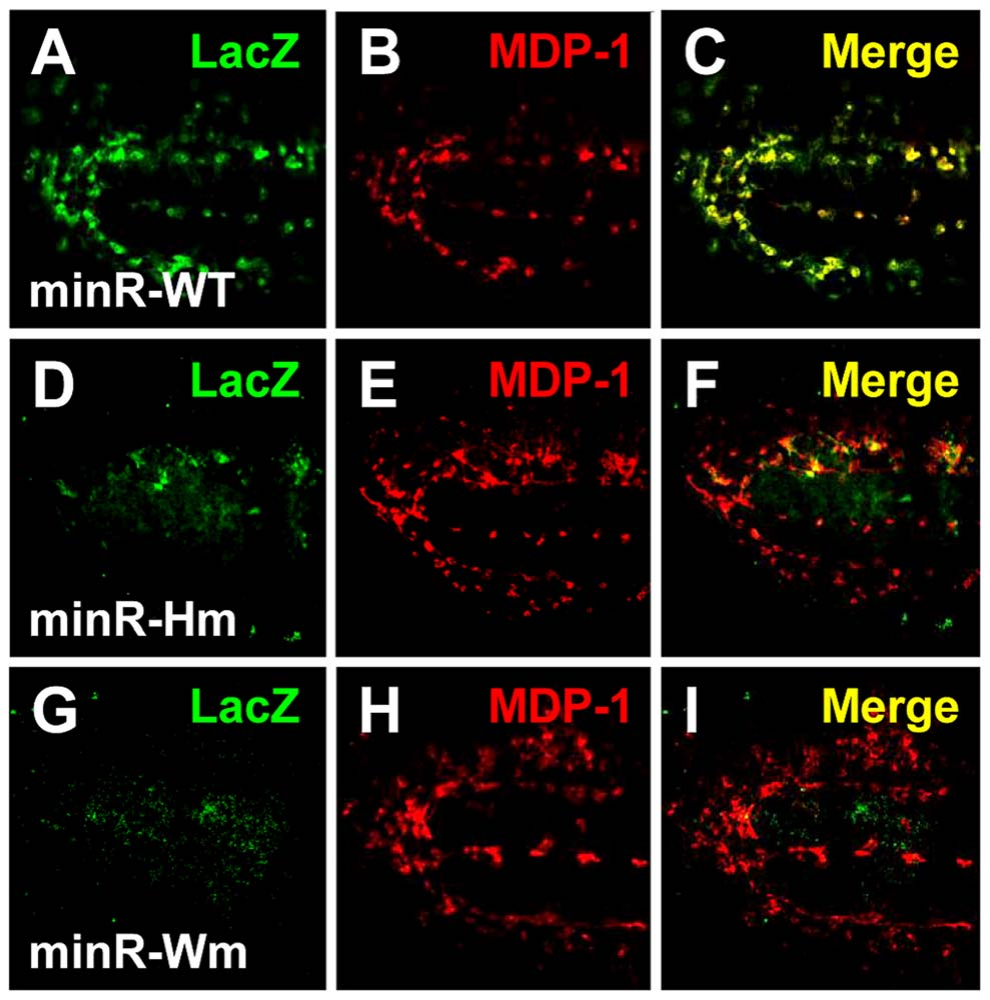
The TCF binding sites are required for expression of the Tig and minR reporters in embryonic hemocytes. (**A–I**) Confocal images of stage 15 embryos containing wild-type minR reporter (A–C) and the Hm (D–F) or Wm (G–I) mutants, with immunofluorescence detection of LacZ (green) and MDP-1 (red). Expression of the reporter is greatly reduced when either motif is mutated.

To confirm that the Tig1 and minR reporters are directly regulated by TCF/Pan in vivo, the WGAWAW sites and r-Helpers in these elements were mutated. Mutation of either motif abolished expression of both reporters in the LG (Figure 11). In embryonic hemocytes, the WGAWAW site mutant of minR had no detectable expression (Figure 12 G–I), while there was some residual hemocytic expression in the r-Helper mutant (Figure 12 D–F). There was no obvious reduction in the Tig1 reporter in embryonic hemocytes when the two functional WGAWAW or two r-Helper sites identified in Kc cells were destroyed (data not shown). This caveat aside, the results indicate that the reverse transcriptional switch documented in Kc cells ([39] and this report) is also operational in the *Drosophila* hematopoietic system.

## Discussion

### Bipartite TCF binding sites mediate a reverse transcriptional switch

This study extends our previous work characterizing WGAWAW sites in the *Ugt36Bc* W-CRM [39], identifying additional sites in another repressed target, *Tig*, and refining the consensus of these sites to RNWGAWAW (Figure 3C). These sites are distinct from traditional HMG sites (SSTTTGWWS) identified in earlier studies of TCF binding [29], [31]. These studies failed to identify WGAWAW sequences as TCF binding sites, perhaps because their experimental designs were biased for the highest affinity sites. However, Badis and coworkers used a microarray of randomized 8-mers to survey DNA binding domains of TFs found WGAWAW sites among the preferred binding sites for HMG domains derived from the four human TCFs [59]. To illustrate this point, we examined where eight functional classic HMG sites from activated W-CRMs and the five WGAWAW sites from the *Tig* and *Ugt36Bc* W-CRMs rank among the nearly 33,000 8-mers tested by Badis and coworkers (Table S1). Two classic sites from a *Notum*/*wingful* W-CRM [33] were the top-ranked site for all four HMG domains, while the third site from this W-CRM ranked 2–4^th^, depending on the protein. For classic sites in two *nkd* W-CRMs [33], [47], the rankings were lower, on average between 112^th^ and 2833^rd^. The repressive WGAWAW sites we identified ranked between 98^th^ and 4167^th^ (Table S1). This work highlights the diversity of DNA recognition by HMG domains (which was also observed for half of the 104 TFs tested in this study) [59], and reveals that WGAWAW sites are a preferred class of HMG binding for TCF/Pan and vertebrate TCFs.

In addition to HMG domain-WGAWAW site binding, we found that C-clamp interactions with r-Helper sites are required for TCF/Pan to regulate the *Tig*, *Ugt36Bc* and minR W-CRMs. The C-clamp is required for regulating the *Ugt36Bc* and *Tig* reporters (Figure 2), and WGAWAW and r-Helper sites in these W-CRMs are required for expression in Kc cells (Figure 4) as well as for the *Tig1* W-CRM in the larval LG (Figure 11). Multimerized WGAWAW-r-Helper site pairs are sufficient for high basal expression and repression by Wnt signaling (Figure 5A, 9 and 11). The three characterized r-Helper sites share a loose consensus of KCCSSNWW and the spacing between adjacent WGAWAW and r-Helper sites is less than 7 bp among the sites we have examined (Figure 3B and S3). More functional WGAWAW, r-Helper site pairs need to be identified to better understand the sequence, spacing and orientation constraints on what constitutes this class of bipartite TCF binding site.

In contrast to the *Ugt36Bc* and *Tig* W-CRMs, in several other cases traditional HMG sites have been found to mediate Wnt repression in *Drosophila* [35], [38] and mammalian cell culture [36], [37]. An examination of the sequences surrounding the functional HMG binding sites in the fly repressed W-CRMs did not reveal obvious candidates for r-Helper or Helper sites (C. Zhang and K. Cadigan, unpublished observations). In these cases, TCF/Pan is proposed to act with other TFs, either competing for binding with an activator [35] or acting in concert with the transcriptional repressor Brinker [38], [60]. We favor the view that the mechanism described in this report is distinct from these other examples of Wnt-mediated repression.

**Figure 13:**
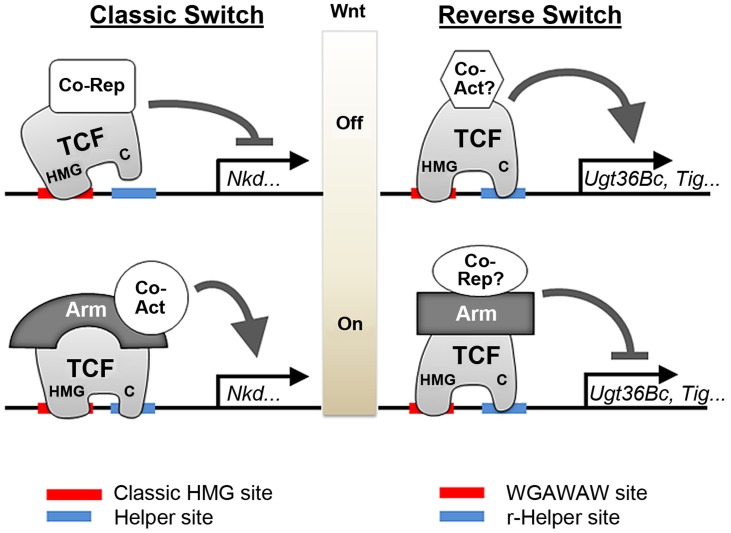
Model for allosteric regulation of TCF/Pan and Arm by bipartite TCF binding sites. The cartoon on the left depicts the classic TCF transcriptional switch, where repression in the absence of Wnt signaling occurs through HMG-HMG site interactions, while Wnt-dependent transcription activation requires DNA binding by both the HMG and C-clamp domains [80]. The cartoon on the right depicts the “reverse transcriptional switch”, where TCF/Pan activates the W-CRM without signaling and represses when complexed with Arm. HMG-WGAWAW site and C-clamp-r-Helper site interactions are required for both sides of the reverse switch. Unknown co-activators and co-repressors are likely to be involved in this regulation. The allosteric regulation of TCF/Pan is represented by different shapes when bound to either class of bipartite binding site; the allostery is likely passed onto other factors such as Arm.

The common models for signal-induced repression require the presence of a default activator bound to DNA near the repressive sites [2], [18], [36]. In contrast, in the TCF-mediated repression described in this report, both basal activation and repression occur through the same TCF binding sites (Figure 13). Mutagenesis of WGAWAW sites and r-Helper sites argue that they are both required for basal activation (Figure 4, 11 and 12), while repression of the minR and Tig reporters by Arm* and DisArmed argue that these sites are also responsible for Wnt-dependent repression (Figure 5, 9 and 10). Consistent with a dual role in regulating these W-CRMs, depletion of TCF/Pan via RNAi resulted in a reduction of basal activation and loss of Wnt-repression (Figure 2). Our data supports the model of a “reverse TCF transcriptional switch” that we have published previously [39], and this work extends this mechanism to the Tig W-CRM and highlights the importance of the C-clamp and r-Helper sites in this regulation (Figure 13).

While we favor the model outlined in Figure 13, it is possible that it is an over-simplification and several things remain to be clarified. For example, mutation of the WGAWAW or r-Helper sites results in a dramatic loss of basal activation (Figure 4, 11 and 12) while depletion of TCF/Pan has a more modest reduction (Figure 2) [39]. This raises the possibility that other TFs could also act through the WGAWAW and r-Helper sites to achieve basal expression. For example, it is possible that TCF/Pan and Arm inhibit transcription by displacing other activating TFs from W-CRM chromatin. Another possibility is that Arm interaction with TCF/Pan disrupts its ability to bind to the bipartite site, though this model is not supported by ChIP data at the *Ugt36Bc* locus [39]. Further investigation is needed to determine whether additional regulators of these W-CRMs exist and if so, how do they functionally interact with TCF/Pan.

### Allosteric regulation of TCF/Pangolin by DNA

Our report provides a dramatic example of how the DNA site can influence the transcriptional output of the TF binding to the site. Replacing classic HMG and Helper sites in a W-CRM (*nkd*-IntE) with low basal expression and a high degree of Wnt activation completely inverted the regulation: the altered W-CRM had high basal expression and was repressed by Wnt signaling (Figure 5B). Just as strikingly, changing 22 bps in the 1.8 kB Tig1 reporter, which converted two WGAWAW and two r-Helper sites into classic motifs, resulted in a W-CRM that behaves like a conventionally activated W-CRM (Figure 5C). Both the HMG and C-clamp binding sites needed to be swapped for this switch in regulation to occur (Figure 5D). These results clearly demonstrate that the type of bipartite TCF binding site to which TCF/Pan binds determines whether it acts as an activator or repressor upon Wnt stimulation.

There are other examples of switching the transcriptional output of CRMs through altering the sequence of TF binding sites. Mutating sequences adjacent to Dorsal binding sites converts a repressed CRM into an activated one, suggesting that for Dorsal, transcriptional activation is the default state [12], [13]. Altering the binding site of Thyroid receptor or POU1 converted CRMs from repressed to activated elements [14], [16], [17]. In these cases, the conversion was only made in one direction, leaving open the possibility that the TF binding sites are not completely sufficient for determining the activation/repression decision.

In our previous report on Wnt mediated TCF/Pan repression, the repressed *Ugt36Bc* W-CRM was converted to an activated one by changing three WGAWAW sites into classic HMG sites [39]. However, Wnt activation was only achieved when the *Ugt36Bc* W-CRM was placed adjacent to the *metallothionein* (*MT*) promoter and a small amount of Cu^2+^ was added [39]. When the *hsp70* promoter was used, the altered *Ugt36Bc* W-CRM was not active, similar to the HMG site only swap in the Tig1 W-CRM (Figure 5D). Our new data strongly suggests that the complications in the prior report were due to our lack of knowledge of Helper sites, which we have now demonstrated to be essential for controlling the transcriptional output of W-CRMs.

The conformation of the HMG and/or C-clamp domains of TCF/Pan is different when bound to a classic HMG-Helper pair compared to a WGAWAW-r-Helper pair, as judged by protease digestion patterns (Figure 6). In addition, the degree of bending of the DNA by the HMG domain is reduced when it is bound to a WGAWAW site (Figure S6). Presumably, these structural differences are transmitted to Arm protein bound to TCF/Pan, leading to differential recruitment of transcriptional co-regulators, as has been suggested for other TFs [20], [61]. Our results add to the growing recognition that TF binding sites are not just for recruiting TFs to regulatory DNA, but also have a profound influence on the TF's functional activity.

### Wnt mediated repression in the hematopoietic system

Repressed W-CRM reporters, either natural (Tig1) or synthetic (minR), are active in embryonic and larval hematopoietic systems (Figure 7, 8 and 10), and are regulated by Wnt signaling (Figure 9 and 10). The data in the LG are especially interesting, given that Wnt signaling has been shown to control several cell fate decisions in this tissue. The Wnt pathway is required for maintenance and proliferation of the posterior signaling center (PSC), which functions as a hematopoietic niche in the LG [40], [62]. In addition, Wnt signaling promotes prohemocytic cell fate, blocking their differentiation in the MZ of the LG as well as promoting proliferation of crystal cells [40]. The Tig and minR reporters displayed minimal expression in the MZ and crystal cells (Figure S7 and S10), and their high expression in the CZ can be repressed by ectopic activation of Arm and DisArmed (Figure 9). Since DisArmed has little/no ability to activate transcription but retains repressive activity [39], these data suggest the existence of Arm-dependent repression of gene expression in the prohemocytes of the MZ.

Wnt-mediated repression of the *Tig* and minR W-CRMs in the LG is likely direct, based on site-directed mutagenesis of the WGAWAW and r-Helper sites (Figure 11 and 12). However, we were unable to demonstrate that endogenous TCF/Pan and Wnt signaling regulates these reporters, because the genetic manipulations also altered the ratio of pro-hemocytes (MZ) and differentiated hemocytes (CZ; Figure S10 and S11). Thus, we could not uncouple cell fate change from regulation of the reporters in our loss of function experiments. It may be that the thresholds for maintaining the CZ and MZ cell fates and regulating the reporters are too similar. Another possibility is that Wnt signaling works redundantly with another factor to repress these reporters in the MZ. Having said this, it's interesting to note that the expression of Peroxidasin (Pxn), normally restricted to the CZ of the LG, expands into the MZ when Wnt signaling is inhibited [40]. Pxn has also been shown to be repressed by Wnt signaling and DisArmed in Kc cells and embryonic hemocytes [39], suggesting a similar relationship in the LG.

The minR synthetic reporter is regulated by Wnt signaling in Kc cells, as well as hemocytes derived from embryos and the LG (Figure 5, 9 and 10). This regulation depends on the WGAWAW and r-Helper sites in all three contexts (Figure 11, 12 and S5). The Tig1 reporter is similarly regulated in Kc cells (Figure 1) and the LG (Figure 9 and 11). In contrast, we found no detectable regulation in embryonic hemocytes (data not shown), even though the reporter is expressed there (Figure 7) and *Tig* transcripts were repressed by Wnt signaling in these cells [39]. We suspect that the 1.8 kb Tig1 reporter may lack some cis-regulatory information required for Wnt regulation in embryonic hemocytes.

Whether the repressive TCF sites can respond to Wnt signaling in other tissues remains unclear, since the minR and Tig reporters have no basal activity outside the hematopoietic system and fat body. To explore whether WGAWAW and r-Helper sites function outside of these tissues, we utilized a GFP reporter containing binding sites for Grainyhead (GRH), which provide basal activity in the imaginal discs [63]. Classic or repressive TCF sites were placed downstream of the GRH sites and transgenic flies generated and analyzed (Figure S12). While classic HMG-Helper site pairs (4TH) displayed strong expression consistent with activation by Wg signaling (Figure S12B, S12G and S12L), insertion of the minR sequences had no effect on the GRH-GFP reporter (Figure S12D, S12I and S12N). These results suggest that WGAWAW and r-Helper sites only respond to Wnt signaling in specific tissues (e.g. the LG). Conversely, 6TH and several other reporters that are activated by Wnt signaling in many tissues [33], [47] are not expressed in the LG (Figure S13). These data argue that the mechanism of Wnt gene regulation in the LG is different from other tissues such as imaginal discs, perhaps because the reverse transcriptional switch mechanism plays a greater role in this tissue. Further studies are needed to identify additional W-CRMs that are active in the LG, and to determine whether the regulatory mechanism uncovered in this report underlies Wnt control of PSC, pro-hemocyte and crystal cell fate in the fly LG.

## Materials and Methods

### *Drosophila* cell culture, RNAi, qRT-PCR, transient transfection and reporter assays

Kc cells were cultured and transient transfections were carried out as previously described [45]. For RNAi treatments, cells were seeded at 1×10^6^ cells/ml in growth media supplemented with 10 µg/ml dsRNA for 4 days, diluted to 1×10^6^ cells/ml without additional dsRNA, and grown for 3 more days for luciferase assay using Tropix Chemiluminescent Kits (Applied Biosystems) or 2 more days for mRNA preparation using Trizol Reagent (Invitrogen). dsRNAs targeting the 3′UTR of *TCF/Pan* [33] and the ORFs of *Axin* or a control gene (*β-lactamase*) were used [39]. qRT–PCR was performed as previously described [39]. Gene expression among different samples was normalized to *tubulin56D* levels.

Each treatment in reporter assays was done in triplicate wells, each containing 2.5×10^5^ cells. For standard reporter assays, 50 ng luciferase reporter and 6.25 ng LacZ per well were transfected with Axin RNAi or control RNAi. For TCF/Pan rescue assays, same amount of reporter and LacZ plus 50 ng TCF/Pan-expressing plasmid and 250 ng Arm* per well were transfected with TCF/Pan RNAi. pAc5.1-V5/His-A vector was used to equalize DNA content between samples and as a negative control for expression vectors. Luciferase activity was normalized to β-galactosidase activity from pArm-LacZ to control for differences in transfection efficiency among samples. In the figures, each bar represents the mean of biological triplicates and the data shown are representative of three independent experiments. All RLA units are arbitrary units unless otherwise specified.

### Plasmids

All luciferase reporter vectors are derivatives of pGL2 or pGL3 (Promega). pHsp-178, Tig1, Tig5 and all site mutants and swaps based on these W-CRMs were cloned into pGL2-basic. Tig2–4, minR, *nkd*-IntE and all site mutants and swaps based on these W-CRMs were cloned into pGL3-basic containing an *Hsp70Bb* minimal promoter. Vector with a *Hsp70Bb* promoter but containing no W-CRM was used to control for basal promoter activity. A *MluI* site was introduced into Tig1 upstream of the TCF sites for the ease of cloning of the swap constructs. Sequence changes were done using site-directed mutagenesis (QuickChange SDM kit, Stratagene) or recursive PCR [64]. Restriction sites and primer sequences are in Table S2 or as previously described [33], [39].

For expression plasmids, pAc-TCF (WT/C-mut), pAc-Arm*, pGEX-GST, pGEX-GST-HMG and pGEX-GST-HMG-C-clamp (WT/C-mut) have been described elsewhere [33], [39]. pArm-LacZ, a derivative of pAc-LacZ (Invitrogen) using the Arm promoter [65] was used as a transfection control.

### EMSA and DNA bending assays

EMSAs were performed as previously described [39]. All GST-tagged proteins used in this study were purified from *E. coli*. 4 nM biotinylated probe (IDT, Coralville, IA) and 7–20 µM protein were used in each reaction. The conditions in the DNA bending assays were similar to the EMSA assays except for the following modifications: 4 nM biotinylated probe was incubated with 20 µM (for WH and WS), 200 nM (for TH) or 500 nM (for TS) protein before separating on 5% native PAGE gel.

The probes for the DNA bending assays were generated according to a previously described strategy [49]. In short, the indicated TCF binding sites were cloned into pGL2-basic vector, and seven pairs of primers at varied positions on the vector were used. PCR products were digested at both ends by *EcoRI*, whose sites were introduced by the primers, and biotinylated through Klenow reaction using Biotin-16-dUTP (Roche). Probes containing a SS site (with both HMG site and Helper site mutated from TH) were generated to confirm that TCF/Pan has no detectable affinity to the surrounding sequences on the probes (Figure S6E). The sequences of TCF binding sites are summarized in Table S2. The WH and WS probes have the binding sites from Tig1 used in the EMSAs shown in Figure 3B. The sequences of the TH, TS and SS sites are previously described [33].

### Fluorescent footprinting

DNaseI fluorescent footprinting was performed as previously described [39]. 20 µM GST-HMG or GST-HMG-C-clamp was used in 50 ul reactions with 12 nM labeled probes. The probes were generated by PCR using one labeled primer and one unlabeled primer (IDT) (Table S2). For comparison between GST and GST-HMG, or GST and GST-HMG-C-clamp, or GST-HMG and GST-HMG-C-clamp, FAM and HEX labeled probes were used in two parallel reactions with different proteins, and combined after digestion. 303 bp in the middle of the *Tig* intronic W-CRM and the full length *Ugt36Bc* W-CRM (178 bp) were footprinted (see Table S2 for sequence information).

### Partial proteolytic digestion and reverse EMSA

20 µl reactions containing 3–6 µM GST-HMG-C-clamp and 20× of the indicated DNA oligonucleotide were incubated for 5 min on ice and 15 min at room temperature. The buffer was the same as used for EMSA but without poly-dI*dC. Protease was then added (for partial proteolytic digestion) or not (for reverse EMSA) at a final concentration of 5–50 ng/µl for chymotrypsin (Roche) or 50–150 ng/µl for endoproteinase Glu-C (New England Biolabs). The mixture was incubated at 25°C for 2.5–3 hours. Then the digested product was loaded onto 16% tricine SDS-PAGE gel [66], and the undigested mixture was loaded onto 6% native PAGE-gel. After running, the gels were silver stained as previously described [67].

### *Drosophila* genetics

Tig (Tig1) and minR fly reporters were generated by cloning the corresponding sequences into pPelican and pHPelican vectors, respectively [50]. All 3×GRH-W-CRM fly reporters were generated by cloning the corresponding sequences into pDestination-eGFP vectors via pENTR/D-TOPO using the Gateway technique, then injecting into integration site 86Fb [68], [69]. Transgenic flies were generated by BestGene Inc. (Chino Hills, CA), Genetic Services Inc. (Cambridge, MA) and Rainbow Transgenic Flies Inc. (Thousand Oaks, California).

All the Gal4 and UAS lines used in this study have been previously described: Srp-Gal4 [70], Dome-Gal4 [71], Lz-Gal4 [72], Cg-Gal4 [73], HmlΔ-Gal4 [74], UAS-Arm* and UAS-DisArmed [39], UAS-Fz^DN^ and UAS-Fz2^DN^ [56], [75] and the DHH triple marker line containing Dome≫EBFP, Hml≫dsRed and Hh≫GFP [76]. The UAS-TCF/Pan-RNAi was a recombinant of two TCF/Pan RNAi lines, one from Vienna Drosophila Resource Center and the other from the *Drosophila* RNAi Screen Center.

The Srp≫Arm* and DisArmed experiments were carried out in the presence of tub-Gal80^ts^. Crosses were set up at 18°C, and the larvae were transferred to 25°C for 2 days (Figure 9) or 3 days (Figure S9) before assaying.

### Immunohistochemistry of embryos and LG

3^rd^-instar larvae were dissected in ice cold PBS from the ventral midline in a similar manner as body wall muscle preparations [43]. For β-galactosidase stainings, exposed LG were fixed in 1% glutaraldehyde at room temperature for 15–20 min, then washed twice and stained in X-gal staining solution [77] with 1–2% X-gal for 10–60 min. Preparation of embryos, immunostaining and microscopy were as previously described, and methods for immunostaining of wing discs were adapted for LG [33]. At least 20 embryos or 12 LGs were analyzed for each condition, and the examples presented are representative.

Primary antibodies were used at the following dilutions: mouse α-wg at 1∶150, mouse α-Cut at 1∶100 and rabbit α-Tig [78] at 1∶75 for LG staining; mouse α-MDP-1 [51] at 1∶100 for embryo staining, and rabbit α-LacZ (MP Biomedicals) at 1∶400 for embryo or 1∶600 for LG staining. Secondary antibodies were described previously [45].

### Immunostaining and quantification of circulating hemocytes

Collection and processing of circulating hemocytes were as described previously [76]. Immunostained circulating hemocytes carrying the minR or Tig1 lacZ reporters were imaged using the Leica SP5 laser scanning confocal microscope with four channels representing LacZ, He≫GFP, P1 (a plasmatocyte marker) [79] and DAPI. Random hemocytes were circled as regions of interest (ROI) and quantified using the Leica LAS AF software. We observed little or no difference between control (He-Gal4≫+) and experimental groups (He≫Arm* or He≫DisArmed) for the DAPI and P1 and some fluctuation in the GFP channel, which could be due to Arm* or DisArmed affecting cell fate/identity. Therefore, we only used hemocytes whose He≫GFP signal intensity falls into the range of control hemocytes. For quantification, 10–15 hemocytes per larvae and 5 larvae per genotype were used.

## Supporting Information

Figure S1 Expression of *Fic* is not affected by Wnt signaling. (**A, B**) Kc cells were treated with control (Wnt Off) or Axin (Wnt On) dsRNA for six days and processed for transcript analysis as described in Materials and Methods. *Tig* expression is repressed by Wnt signaling (A), which *Fic* expression is unaffected (B). (**C, D**) Mutations in r-Helper sites (H) or WGAWAW sites (W) greatly decrease the basal activity and repression of the *Tig* and *Ugt36Bc* W-CRM reporters in Kc cells by Arm* expression. *p<0.05; n.s., not significant (Student's T-test).
(TIF)

Figure S2 Sequences protected by GST-HMG and/or GST-HMG-C-clamp in the *Tig* intron. (**A**) The 200 bp stretch of the *Tig* probe containing all footprinted regions is shown, with the HMG domain and C-clamp protected regions indicated. Two WGAWAW sites (red) are bound by the HMG domain, as well as several other sites (green). r-Helper sites bound by the C-clamp are shown in blue. The sequences that were mutated for the reporter assays shown in Figure 4 or Figure S2B are indicated with asterisks. (**B**) Tig1 reporters containing mutations in the TG-rich regions footprinted by the HMG domain were similar to the wild-type control.
(TIF)

Figure S3 Footprinting chromatographs showing the C-clamp-specific protection of the r-Helper sites in the *Tig* W-CRM. Regions where the blue signals are higher than the green signals were protected by GST-HMG-C-clamp and not by GST-HMG. Note that the arbitrary colors are switched compared to those shown in Figure 3B.
(TIF)

Figure S4 Sequence information of minR and *nkd*-IntE and Tig1 “swapped site” reporters. For all constructs, classic HMG binding and WGAWAW sites shown in red, while Helper and r-Helper sites shown in blue. (**A**) minR W-CRM and variations with the WGAWAW or r-Helper sites mutated (altered nucleotides in lower case). (**B**) The entire 255 bp *nkd*-IntE W-CRM, with sites to be swapped underlined and the sequence of the W-CRM with classic sites converted into WGAWAW and r-Helper sites. (**C**) Portion of the *Tig* first intron containing the two functional WGAWAW and r-Helper sites, plus the sequences where these motifs are swapped into sites typical of activated W-CRMs. The altered nucleotides in the swapped reporters are shown in lowercase.
(TIF)

Figure S5 The activity of minR is dependent on r-Helper and WGAWAW sites. (**A**) Similar to Tig1, the minR reporter is repressed by Arm* expression. (B) When either r-Helper or WGAWAW sites were mutated, the basal activity of minR reporter and its response to Wnt signaling (Axin RNAi) were both strongly decreased. *p<0.05; ***p<0.001; n.s.: not significant (Student's T-test).
(TIF)

Figure S6 DNA bending by the HMG domain of TCF/Pan. (**A**) Cartoon showing a series of seven probes, each with a bipartite TCF binding site (red/blue boxes) located along the 139 bp oligonucleotide. These TCF sites could consist of a classic HMG or WGAWAW site (TS or WS) or HMG-Helper or WGAWAW-r-Helper pair (TH or WH). If DNA bending occurs upon protein binding, the complex will run slower in an EMSA when the binding site is in the middle of the probe [49]. (**B**) GST-HMG protein bends TS slightly more than WS. (**C**) The presence of a Helper site does not increase the bending observed when GST-HMG-C-clamp binds to a HMG site. (**D**) GST-HMG-C-clamp bends TH slightly more than WH. (**E**) Probes with the identical spacer sequences as the TS, WS, TH and WH probes but lacking HMG and Helper sites (SS) were not bound by GST-HMG-C-clamp. Two SS probes, corresponding to the fourth and seventh probes in the series of seven probes (A), were tested. Each experiment was performed at least three times with similar results.
(TIF)

Figure S7 The Tig and minR reporters are not active in crystal cells. (**A–H**) Larval LGs from late 3^rd^ instar larvae containing p[Lz-Gal4] and p[UAS-mCD8::GFP] and the minR (A–D) or Tig1 (E–H) lacZ reporters, with LacZ immunodetection (red). Both fluorescent signals are cytosolic. Panels B–D and F–H are higher magnification of the boxed regions in A and E, respectively. The expression patterns of the reporters are largely exclusive with Lz≫GFP, a marker of crystal cells which often express Wg [40].
(TIF)

Figure S8 Cut immunostaining marks the CZ of the larval LG. Cut (red) colocalizes with Cg≫GFP (**A–F**) and Hml≫GFP (green) (**G–I**), two established CZ markers [53], [76]. Multiple glands are shown to recapitulate the variation in the shape of CZ/MZ. Cut is a nuclear protein, while Hml≫GFP signal is cytosolic and Cg≫mCD8::GFP is localized to the membrane.
(TIF)

Figure S9 Activation of Wnt signaling in the larval LG is able to affect cell fate and reduce the size of the CZ. (**A–F**) Micrographs of older 3^rd^ instar larval LGs from strains containing the minR reporter and P[Srp-Gal4], without (A–C) or with P[UAS-Arm*] (D–F). Activation of Wnt signaling by Arm* expression greatly reduces the size of CZ, indicated by Cut (red), and expression of the lacZ reporter (green) is greatly reduced.
(TIF)

Figure S10 Inhibition of Wnt signaling by Fz^DN^ and Fz2^DN^ in the MZ derepresses minR signal but also reduces the size of MZ. (**A–F**) Micrographs of younger late 3^rd^ instar larval LGs (∼94–98 hr AEL) from strains containing the minR reporter, P[UAS-mCD8::GFP] and P[Dome-Gal4] without (A–C) or with P[Fz^DN^; Fz2^DN^] (D–F). Dome≫GFP indicates MZ cells, while lacZ positive cells are in the CZ. Inhibition of Wnt signaling by Fz^DN^ and Fz2^DN^ expression increases lacZ reporter signal (red) in the CZ, but also reduces GFP expression, indicating an decrease in the size of the MZ.
(TIF)

Figure S11 TCF/Pan knockdown in the CZ reduces minR expression but also reduces the size of the CZ. (**A–F**) Micrographs of older late 3^rd^ instar larval LGs from strains containing the minR reporter, P[UAS-mCD8::GFP] and P[Hml-Gal4], without (A–C) or with P[UAS-TCF/Pan-RNAi] (D–F). Depletion of TCF reduces minR reporter expression (red), but also reduces the GFP signal indicating a reduction in the size of the CZ.
(TIF)

Figure S12 WGAWAW, r-Helper site pairs do not affect transcription in several other tissues outside the hematopoietic system. (**A**) A cartoon showing the structure of 3×GRH-W-CRM reporters, containing three Grainyhead (GRH) binding sites which provides basal activity in the tissues being tested and a W-CRM followed by a EGFP reporter gene. (**B–P**) Micrographs of wing (B–F), leg (G–K) and eye-antenna (L–P) discs from 3^rd^ instar larvae carrying indicated 3×GRH-W-CRMs. 3×GRH-4TH contains four classic HMG-Helper site pairs, and displays high expression in regions where Wg is known to be expressed. 3×GRH-SS contains randon sequences and has the low level, ubiquitous pattern previously described [63]. 3×GRH-minR-WT along with the r-Helper (Hm) and WGAWAW (Wm) site mutant versions are all expressed in very similar patterns to 3×GRH-SS, with no hint of basal activation or Wg-dependent repression. (**Q**) Sequence information for the 3×GRH-W-CRM reporters.
(TIF)

Figure S13 Several Wnt-activated W-CRMs have no detectable activity in the LG. (**A–E**) Comparison between minR and Wnt-activated reporters stained with X-gal. The minR reporter (B) shows strong staining in the CZ, while all the Wnt-activated W-CRMs tested (C–E), as well as the negative control w^1118^ (A), have no detectable staining. The minR reporter was stained for the same amoun of time as the other reporter lines, resulting in over-staining. (**F**) Micrograph of an older 3^rd^ instar larval LG stained with X-gal, taken with DIC optics, highlighting the larger, less densely packed cells of the CZ. (**G**) Brightfield image of the same LG where the LacZ staining is more pronounced. (**H**) Brightfield image of the same LG where the DIC image was used to draw a broken white line separating the CZ and MZ.
(TIF)

Table S1 Preference of WGAWAW binding by the HMG domains of vertebrate TCFs, taken from Badis et al. (2009). HMG binding sites from five repressed and eight activated W-CRMs were analyzed [33], [39], [47]. Numbers represent ranking out of a pool of 32896 8-mers tested by Badis and co-workers [59]. Two data sets from each TCF family member are shown (#1 and #2). The underlined sequence denotes the HMG binding site from each W-CRM within a specifc 8-mer. These sequences are found in more than one 8-mer; the ones with the highest ranking are shown and the highest ranking 8-mer containing each binding sequence is highlighted in yellow. While the sites from the Notum/wingful W-CRM are found in the highest ranked 8-mers, the range for the other sites from activated W-CRMs are similar to those found in repressed W-CRMs.
(XLSX)

Table S2 Oligonucleotide sequences used in this paper. Note that the sequences for DNA bending assay were presented in a longer probe (see Materials and Methods for more information).
(XLSX)
