# Supplementary figures and images for: Wnt-Mediated Repression via Bipartite DNA Recognition by TCF in the Drosophila Hematopoietic System
Source: PLoS Genet. 2014 Aug 21;10(8):e1004509. doi: 10.1371/journal.pgen.1004509 (PMC4140642; doi:10.1371/journal.pgen.1004509)

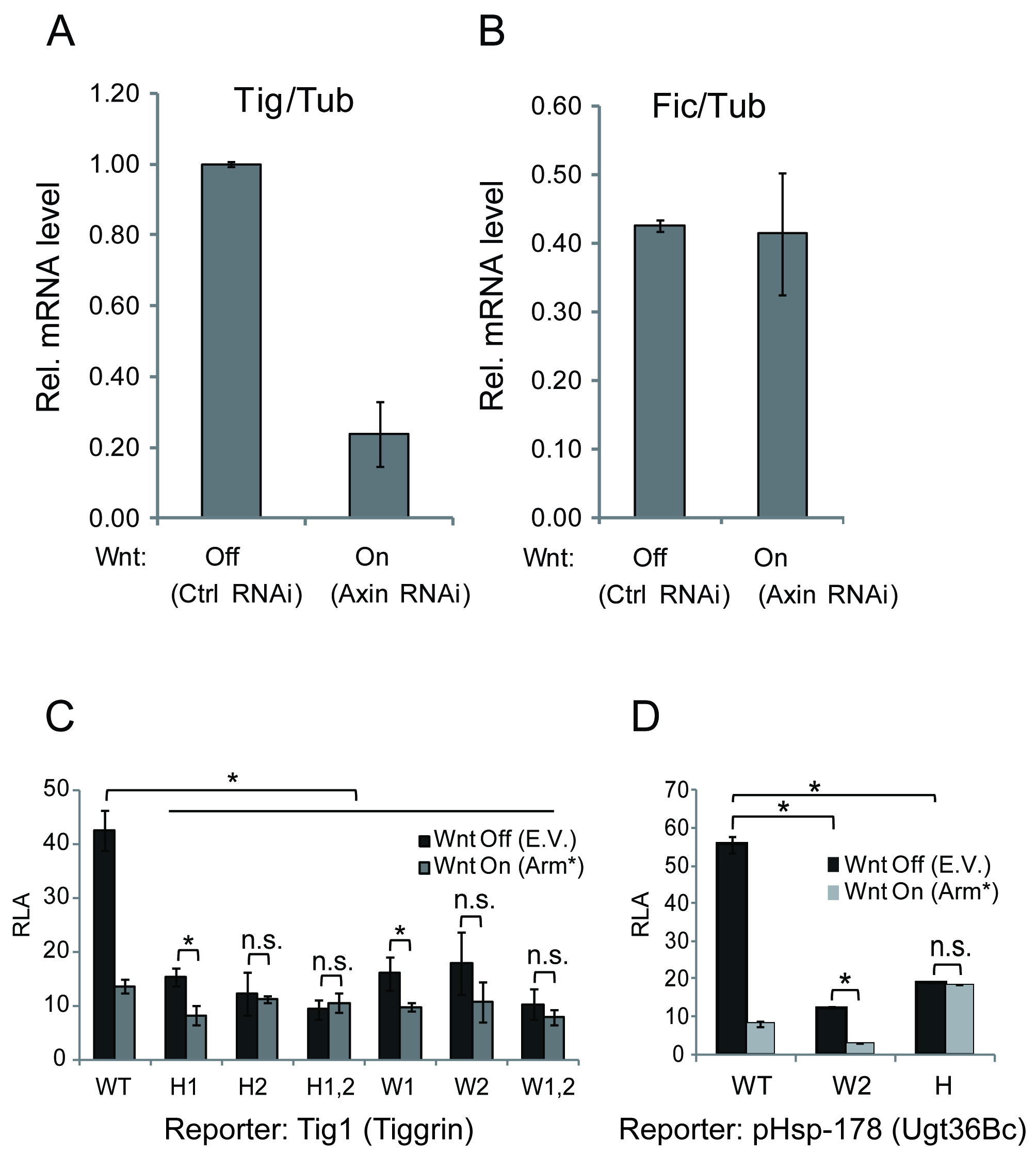

Supplement: Figure S1 — Expression of Fic is not affected by Wnt signaling. (A, B) Kc cells were treated with control (Wnt Off) or Axin (Wnt On) dsRNA for six days and processed for transcript analysis as described in Materials and Methods. Tig expression is repressed by Wnt signaling (A), which Fic expression is unaffected (B). (C, D) Mutations in r-Helper sites (H) or WGAWAW sites (W) greatly decrease the basal activity and repression of the Tig and Ugt36Bc W-CRM reporters in Kc cells by Arm* expression. *p<0.05; n.s., not significant (Student's T-test). (TIF) [file pgen.1004509.s001.tif]

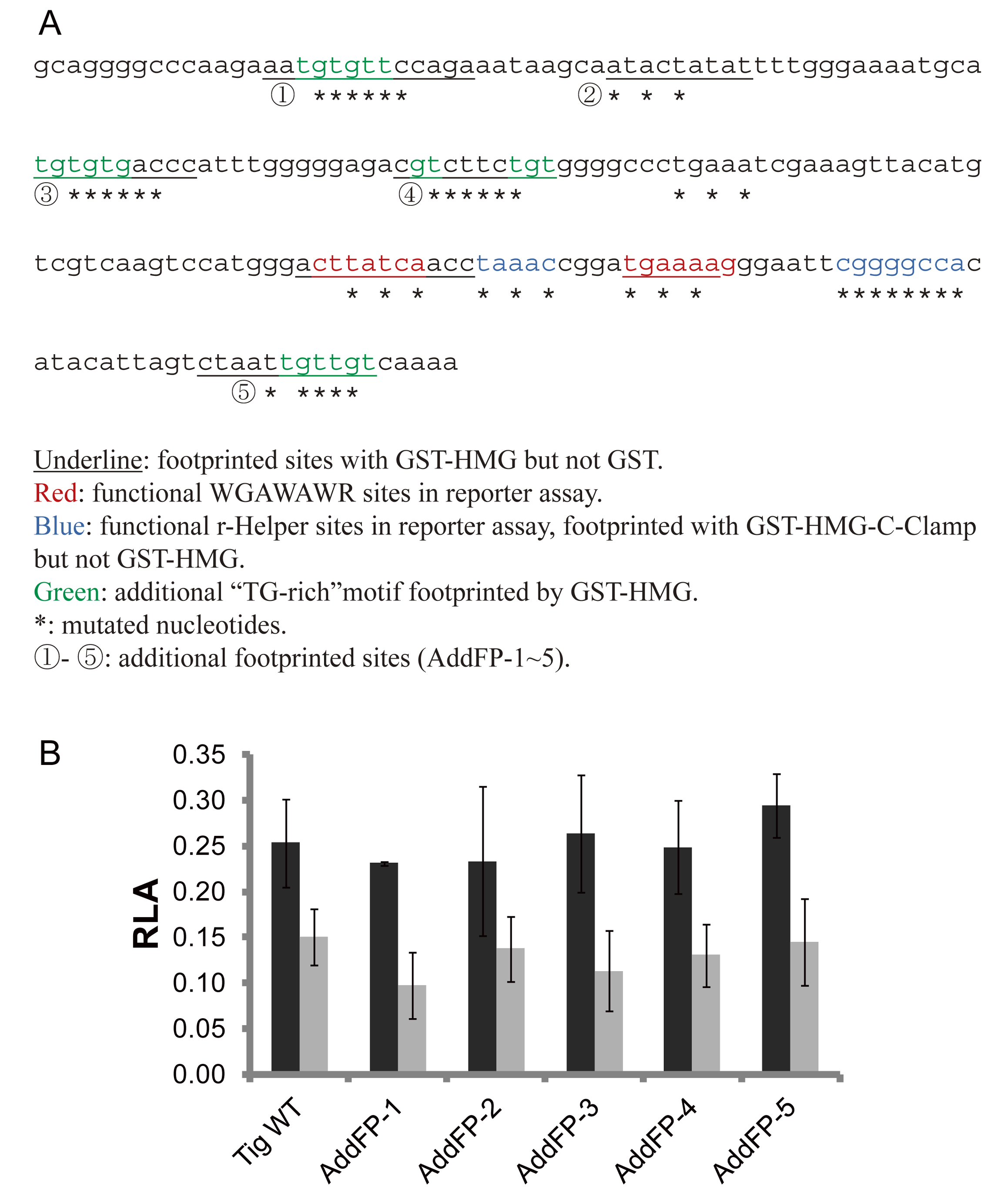

Supplement: Figure S2 — Sequences protected by GST-HMG and/or GST-HMG-C-clamp in the Tig intron. (A) The 200 bp stretch of the Tig probe containing all footprinted regions is shown, with the HMG domain and C-clamp protected regions indicated. Two WGAWAW sites (red) are bound by the HMG domain, as well as several other sites (green). r-Helper sites bound by the C-clamp are shown in blue. The sequences that were mutated for the reporter assays shown in Figure 4 or Figure S2B are indicated with asterisks. (B) Tig1 reporters containing mutations in the TG-rich regions footprinted by the HMG domain were similar to the wild-type control. (TIF) [file pgen.1004509.s002.tif]

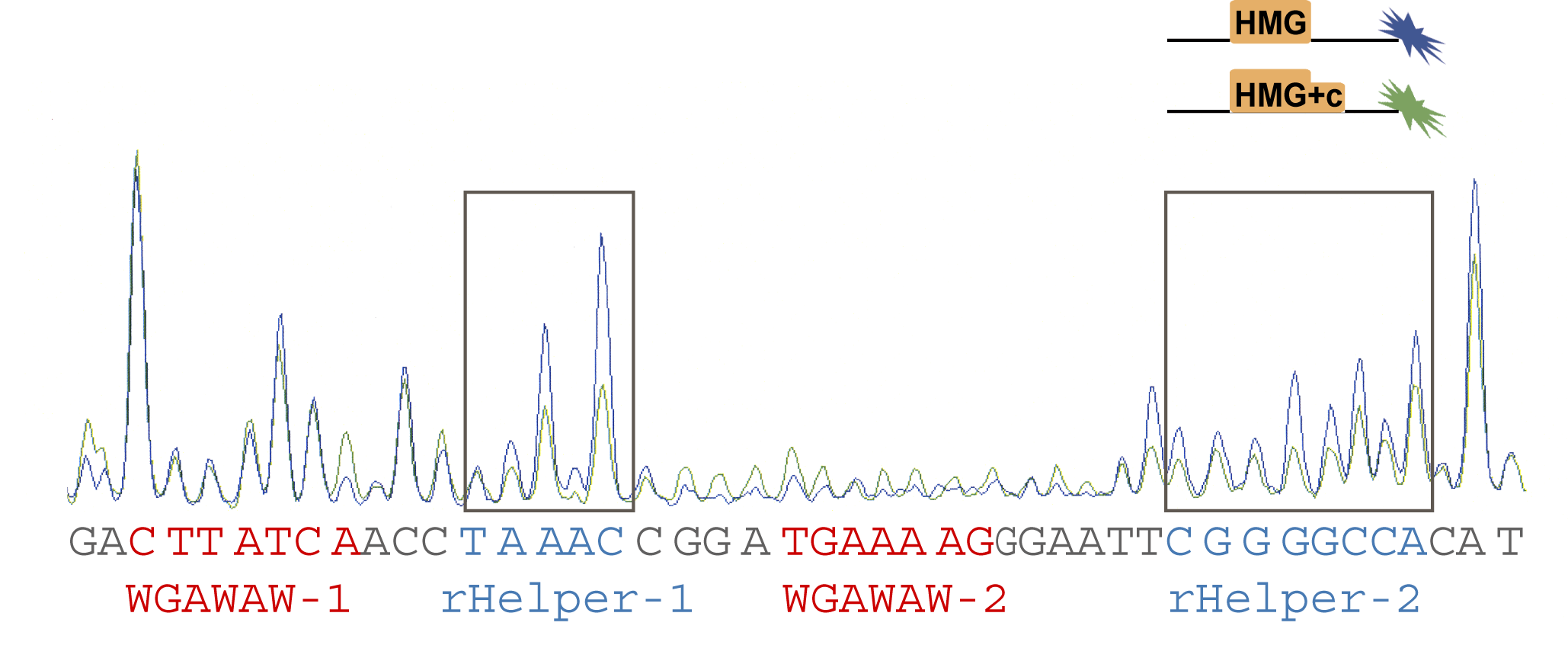

Supplement: Figure S3 — Footprinting chromatographs showing the C-clamp-specific protection of the r-Helper sites in the Tig W-CRM. Regions where the blue signals are higher than the green signals were protected by GST-HMG-C-clamp and not by GST-HMG. Note that the arbitrary colors are switched compared to those shown in Figure 3B. (TIF) [file pgen.1004509.s003.tif]

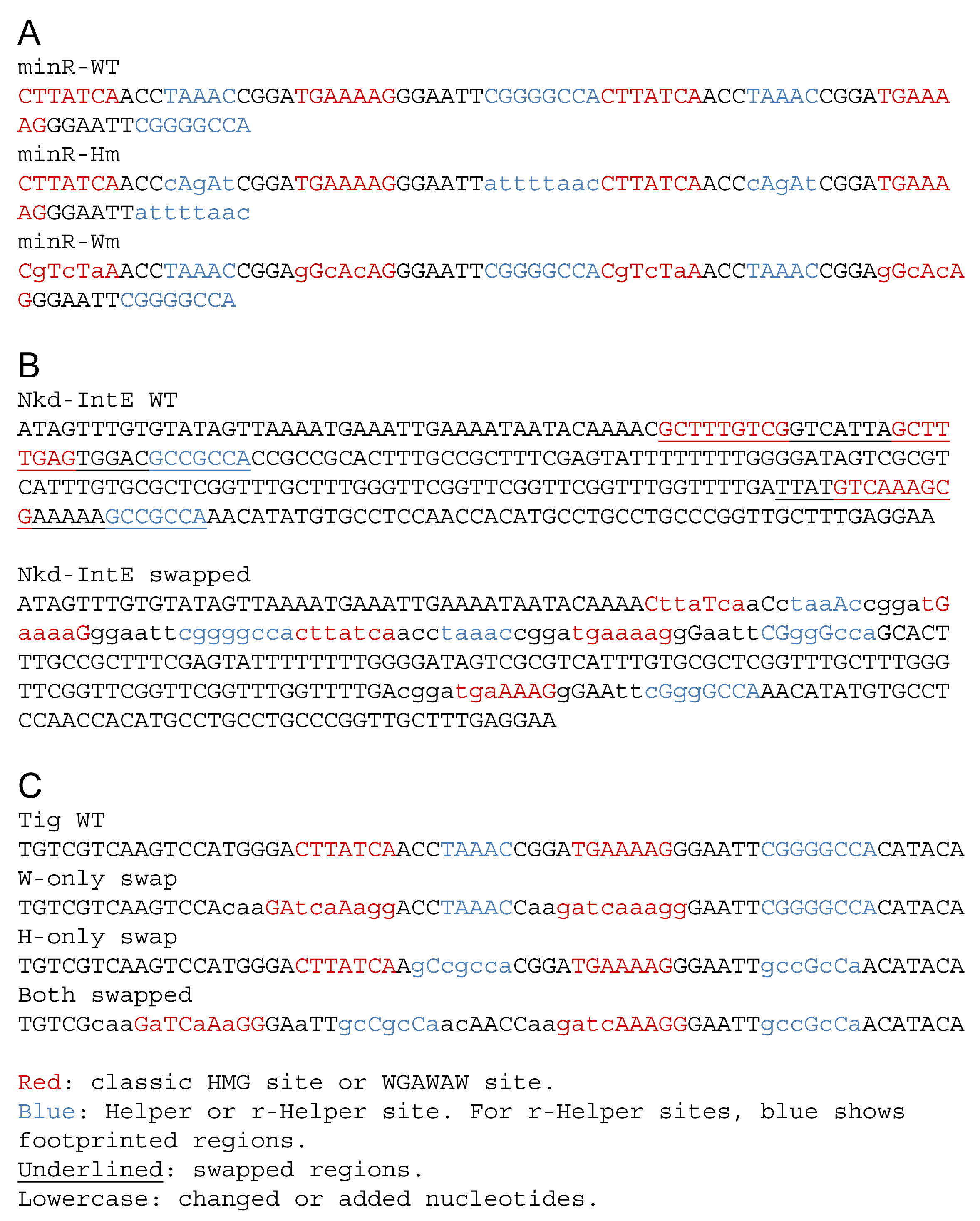

Supplement: Figure S4 — Sequence information of minR and nkd-IntE and Tig1 “swapped site” reporters. For all constructs, classic HMG binding and WGAWAW sites shown in red, while Helper and r-Helper sites shown in blue. (A) minR W-CRM and variations with the WGAWAW or r-Helper sites mutated (altered nucleotides in lower case). (B) The entire 255 bp nkd-IntE W-CRM, with sites to be swapped underlined and the sequence of the W-CRM with classic sites converted into WGAWAW and r-Helper sites. (C) Portion of the Tig first intron containing the two functional WGAWAW and r-Helper sites, plus the sequences where these motifs are swapped into sites typical of activated W-CRMs. The altered nucleotides in the swapped reporters are shown in lowercase. (TIF) [file pgen.1004509.s004.tif]

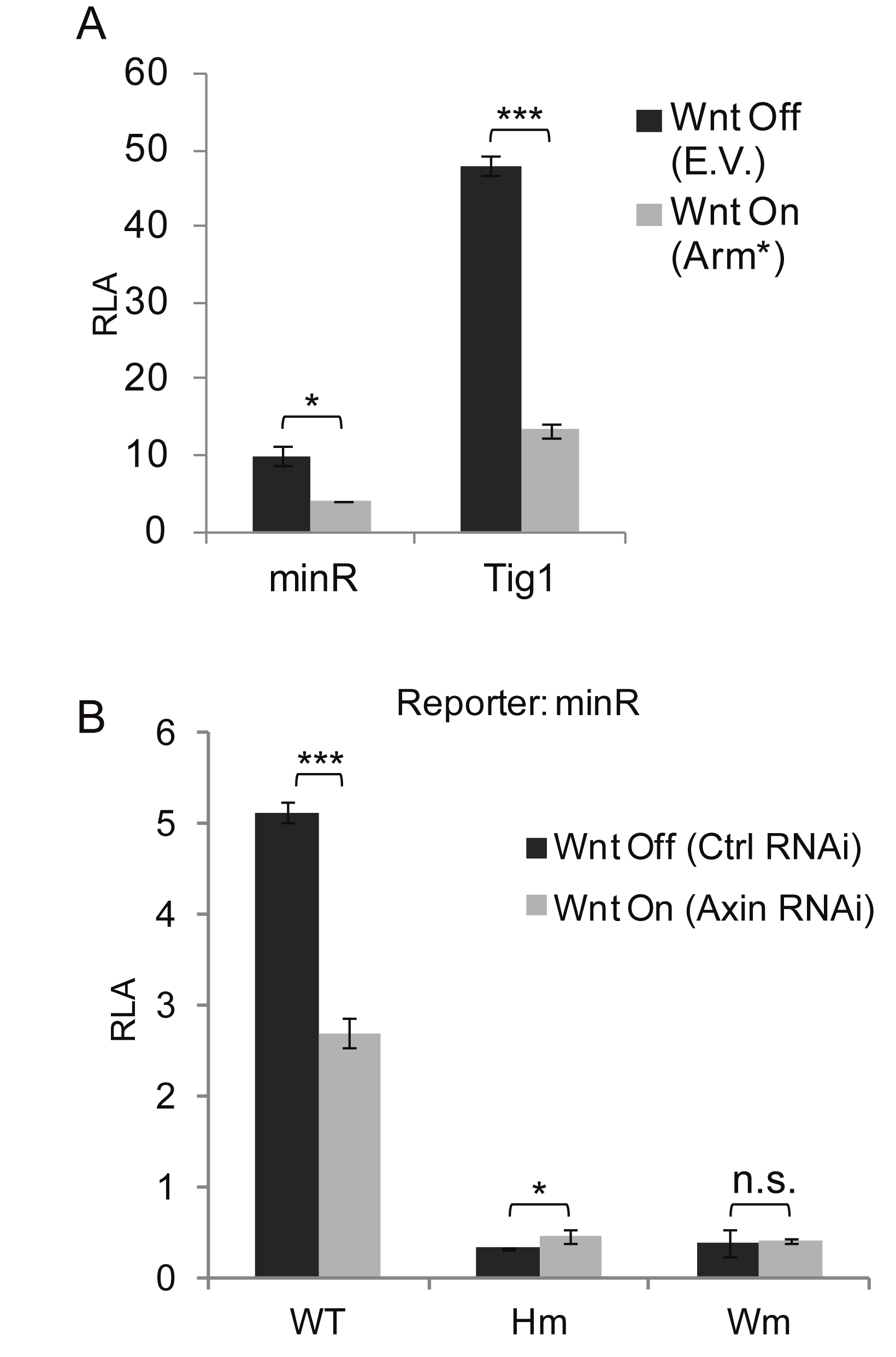

Supplement: Figure S5 — The activity of minR is dependent on r-Helper and WGAWAW sites. (A) Similar to Tig1, the minR reporter is repressed by Arm* expression. (B) When either r-Helper or WGAWAW sites were mutated, the basal activity of minR reporter and its response to Wnt signaling (Axin RNAi) were both strongly decreased. *p<0.05; ***p<0.001; n.s.: not significant (Student's T-test). (TIF) [file pgen.1004509.s005.tif]

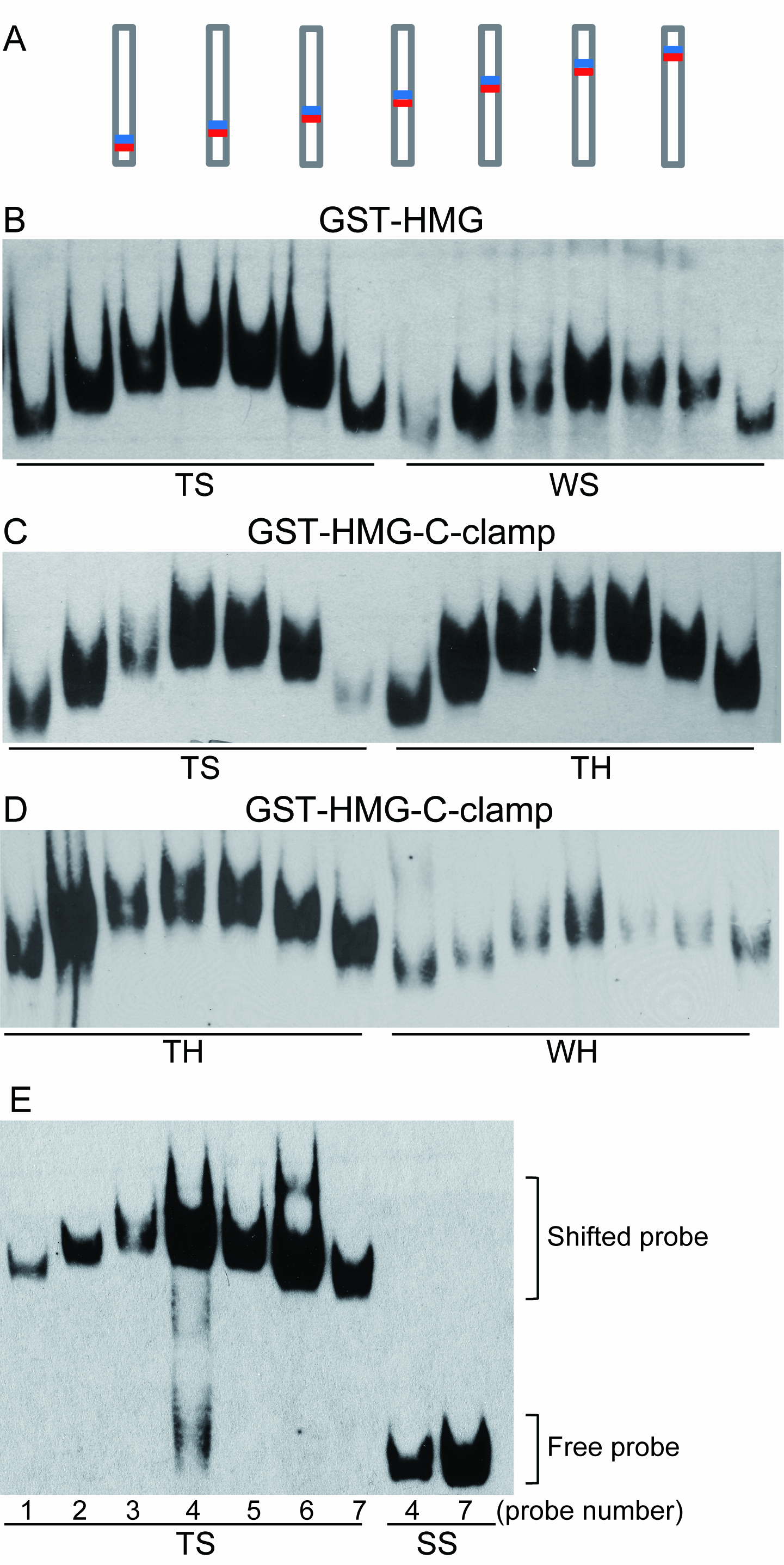

Supplement: Figure S6 — DNA bending by the HMG domain of TCF/Pan. (A) Cartoon showing a series of seven probes, each with a bipartite TCF binding site (red/blue boxes) located along the 139 bp oligonucleotide. These TCF sites could consist of a classic HMG or WGAWAW site (TS or WS) or HMG-Helper or WGAWAW-r-Helper pair (TH or WH). If DNA bending occurs upon protein binding, the complex will run slower in an EMSA when the binding site is in the middle of the probe [49]. (B) GST-HMG protein bends TS slightly more than WS. (C) The presence of a Helper site does not increase the bending observed when GST-HMG-C-clamp binds to a HMG site. (D) GST-HMG-C-clamp bends TH slightly more than WH. (E) Probes with the identical spacer sequences as the TS, WS, TH and WH probes but lacking HMG and Helper sites (SS) were not bound by GST-HMG-C-clamp. Two SS probes, corresponding to the fourth and seventh probes in the series of seven probes (A), were tested. Each experiment was performed at least three times with similar results. (TIF) [file pgen.1004509.s006.tif]

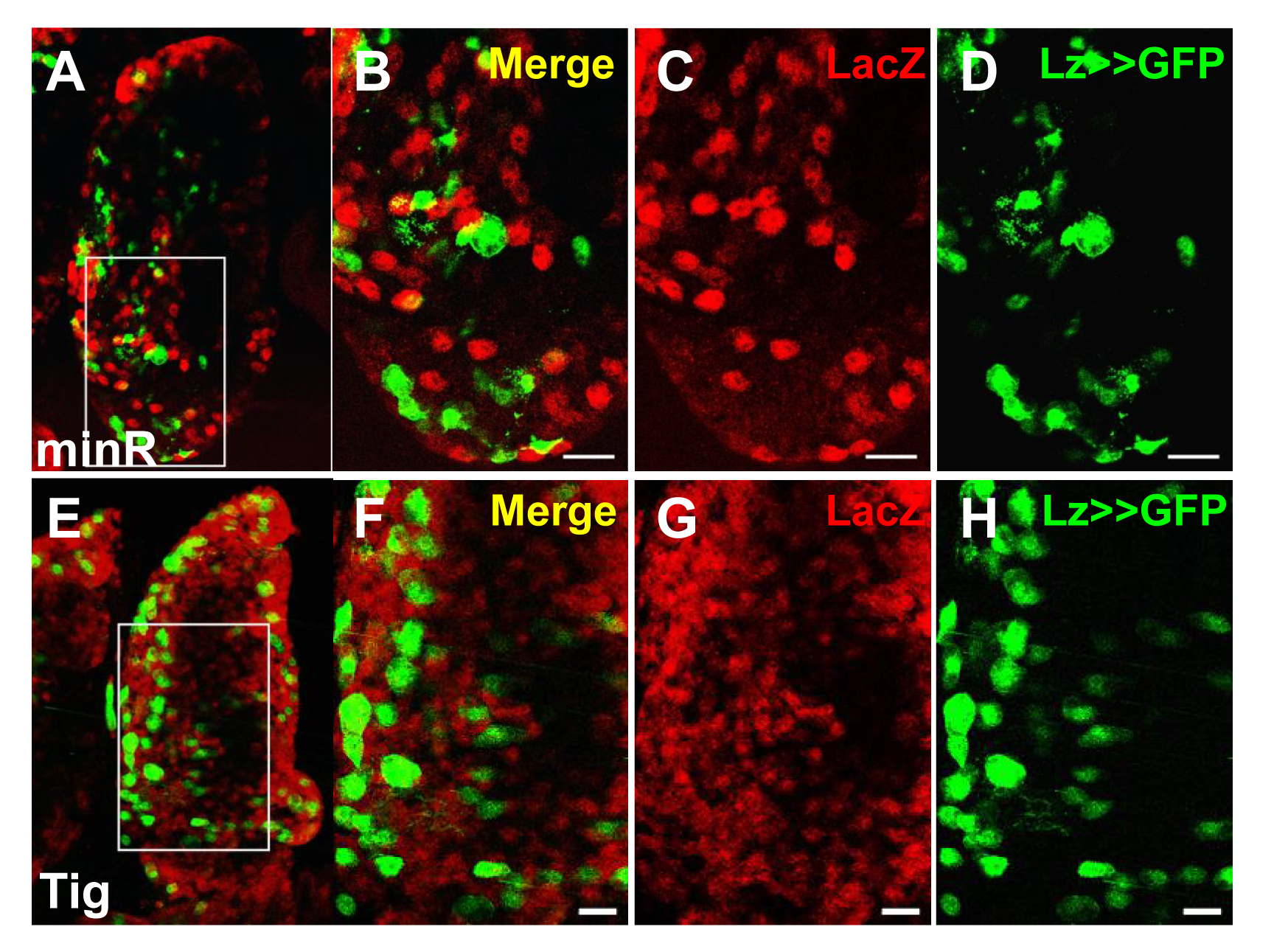

Supplement: Figure S7 — The Tig and minR reporters are not active in crystal cells. (A–H) Larval LGs from late 3rd instar larvae containing p[Lz-Gal4] and p[UAS-mCD8::GFP] and the minR (A–D) or Tig1 (E–H) lacZ reporters, with LacZ immunodetection (red). Both fluorescent signals are cytosolic. Panels B–D and F–H are higher magnification of the boxed regions in A and E, respectively. The expression patterns of the reporters are largely exclusive with Lz≫GFP, a marker of crystal cells which often express Wg [40]. (TIF) [file pgen.1004509.s007.tif]

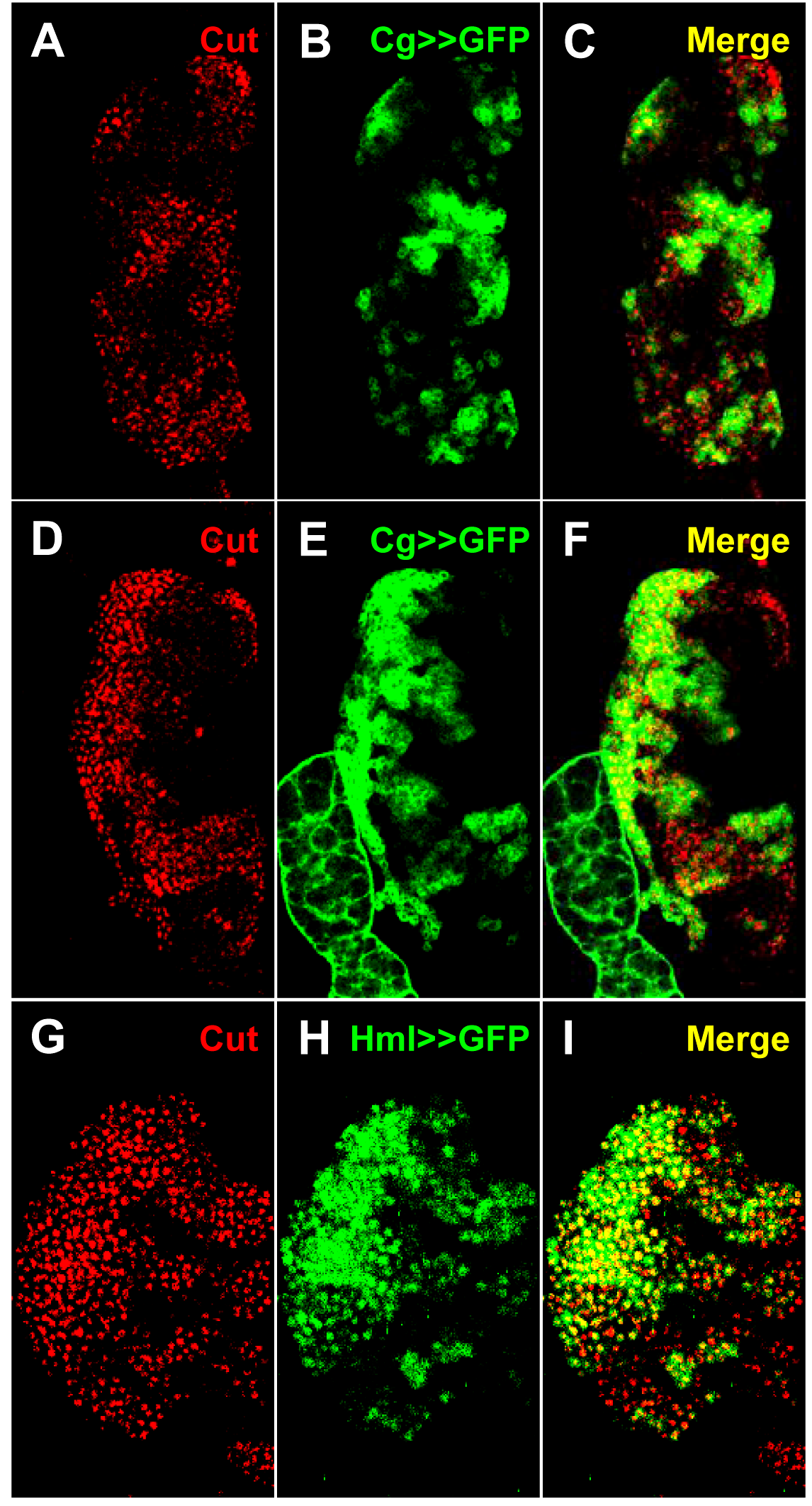

Supplement: Figure S8 — Cut immunostaining marks the CZ of the larval LG. Cut (red) colocalizes with Cg≫GFP (A–F) and Hml≫GFP (green) (G–I), two established CZ markers [53], [76]. Multiple glands are shown to recapitulate the variation in the shape of CZ/MZ. Cut is a nuclear protein, while Hml≫GFP signal is cytosolic and Cg≫mCD8::GFP is localized to the membrane. (TIF) [file pgen.1004509.s008.tif]

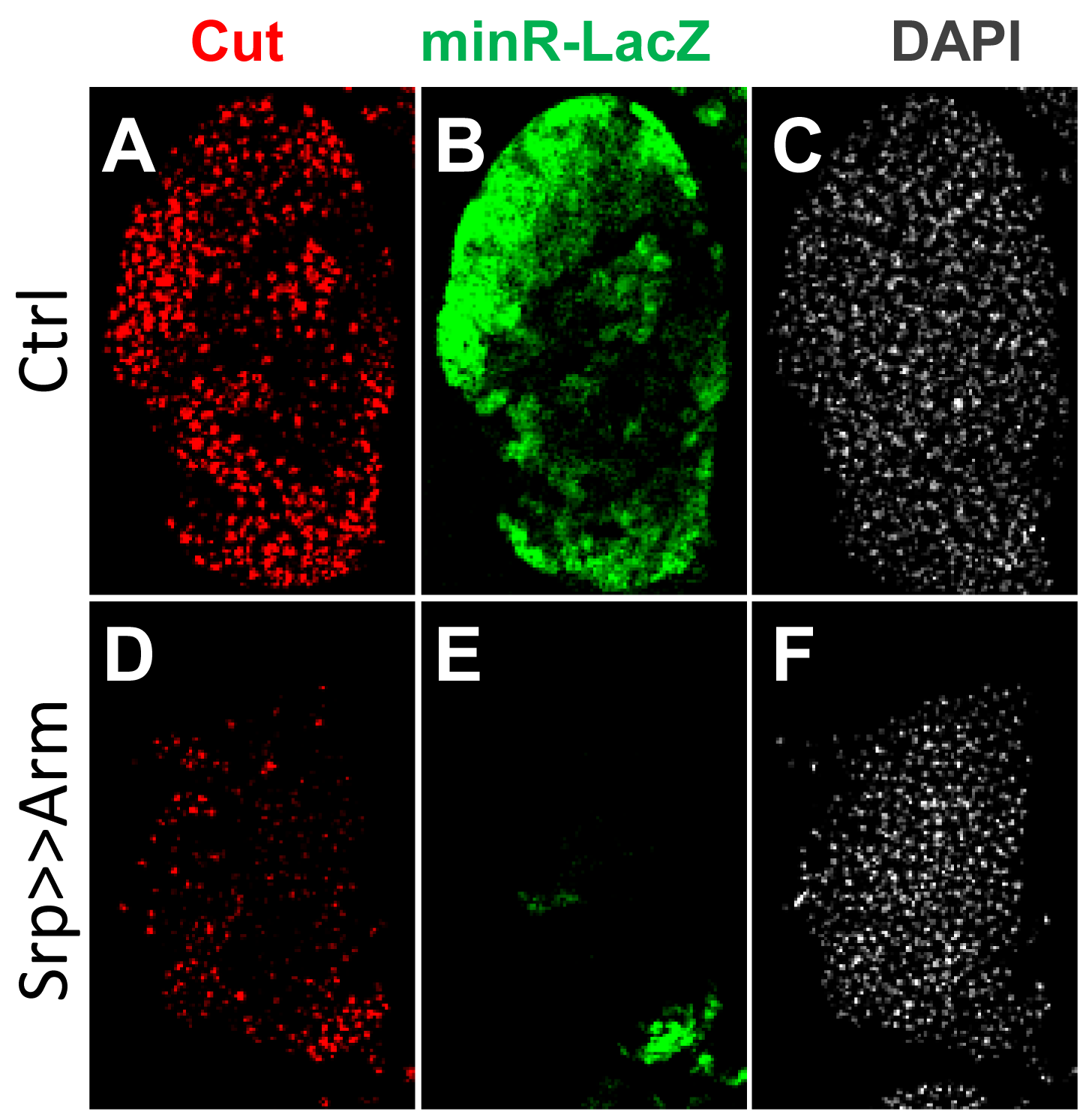

Supplement: Figure S9 — Activation of Wnt signaling in the larval LG is able to affect cell fate and reduce the size of the CZ. (A–F) Micrographs of older 3rd instar larval LGs from strains containing the minR reporter and P[Srp-Gal4], without (A–C) or with P[UAS-Arm*] (D–F). Activation of Wnt signaling by Arm* expression greatly reduces the size of CZ, indicated by Cut (red), and expression of the lacZ reporter (green) is greatly reduced. (TIF) [file pgen.1004509.s009.tif]

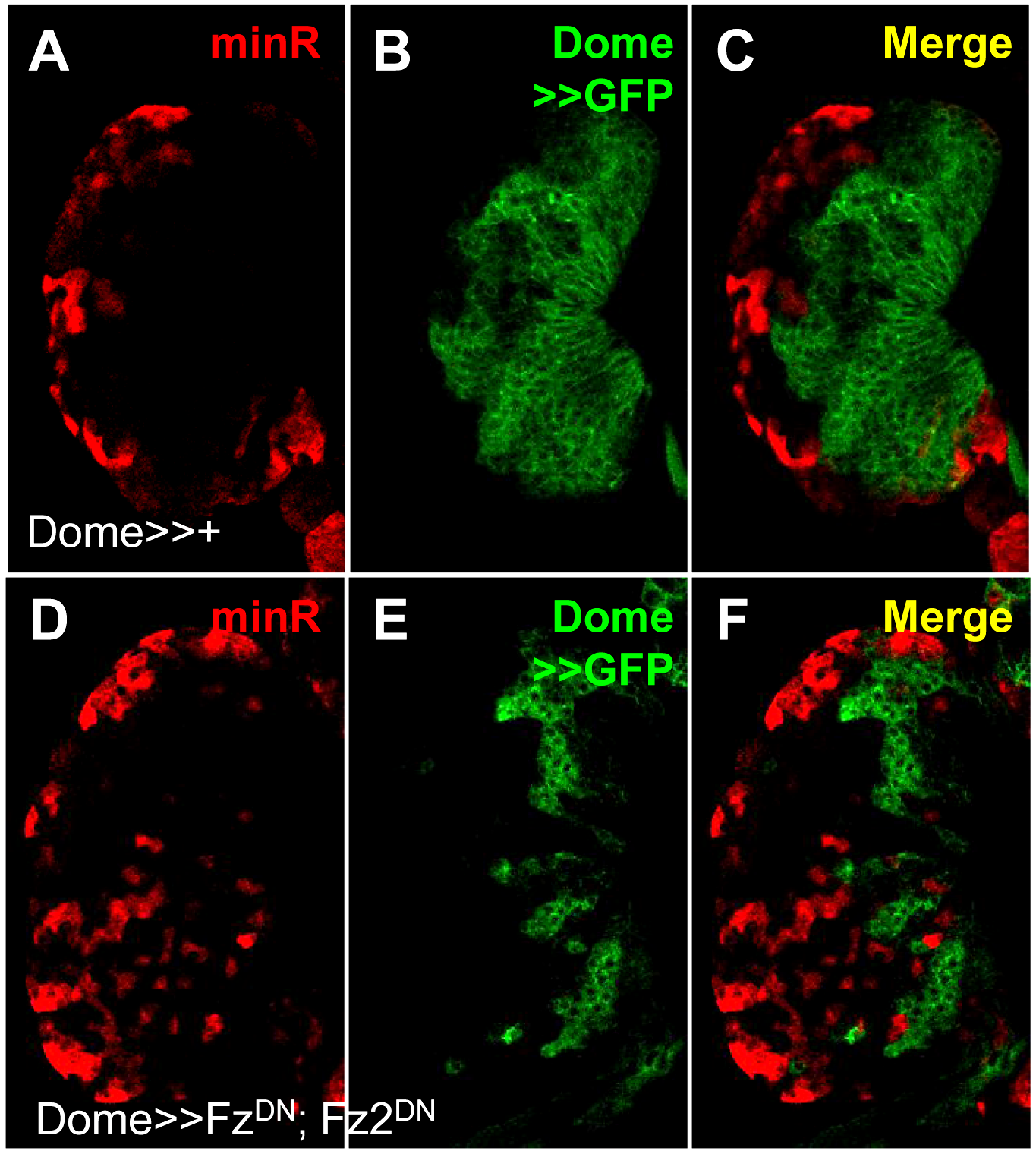

Supplement: Figure S10 — Inhibition of Wnt signaling by FzDN and Fz2DN in the MZ derepresses minR signal but also reduces the size of MZ. (A–F) Micrographs of younger late 3rd instar larval LGs (∼94–98 hr AEL) from strains containing the minR reporter, P[UAS-mCD8::GFP] and P[Dome-Gal4] without (A–C) or with P[FzDN; Fz2DN] (D–F). Dome≫GFP indicates MZ cells, while lacZ positive cells are in the CZ. Inhibition of Wnt signaling by FzDN and Fz2DN expression increases lacZ reporter signal (red) in the CZ, but also reduces GFP expression, indicating an decrease in the size of the MZ. (TIF) [file pgen.1004509.s010.tif]

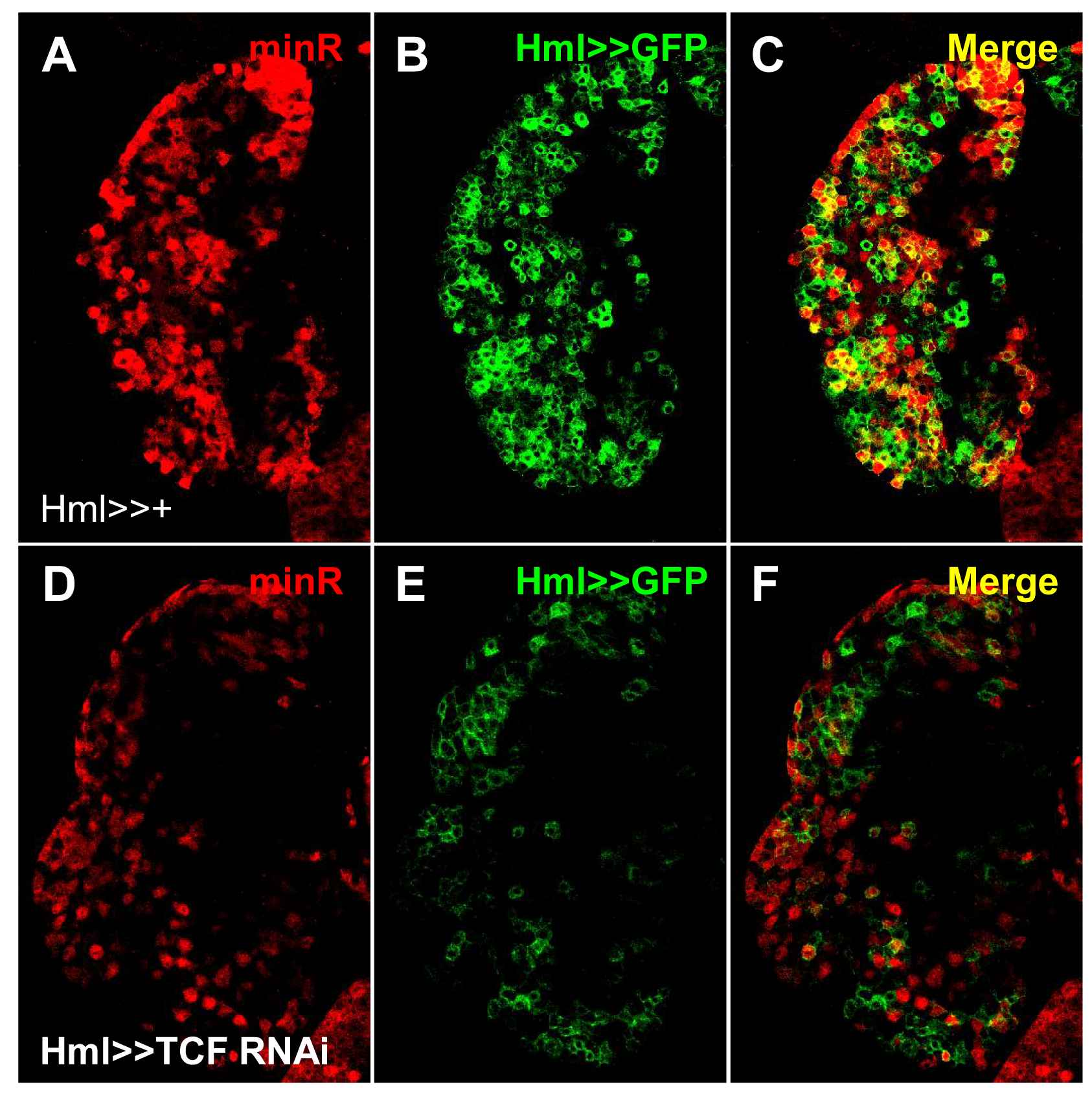

Supplement: Figure S11 — TCF/Pan knockdown in the CZ reduces minR expression but also reduces the size of the CZ. (A–F) Micrographs of older late 3rd instar larval LGs from strains containing the minR reporter, P[UAS-mCD8::GFP] and P[Hml-Gal4], without (A–C) or with P[UAS-TCF/Pan-RNAi] (D–F). Depletion of TCF reduces minR reporter expression (red), but also reduces the GFP signal indicating a reduction in the size of the CZ. (TIF) [file pgen.1004509.s011.tif]

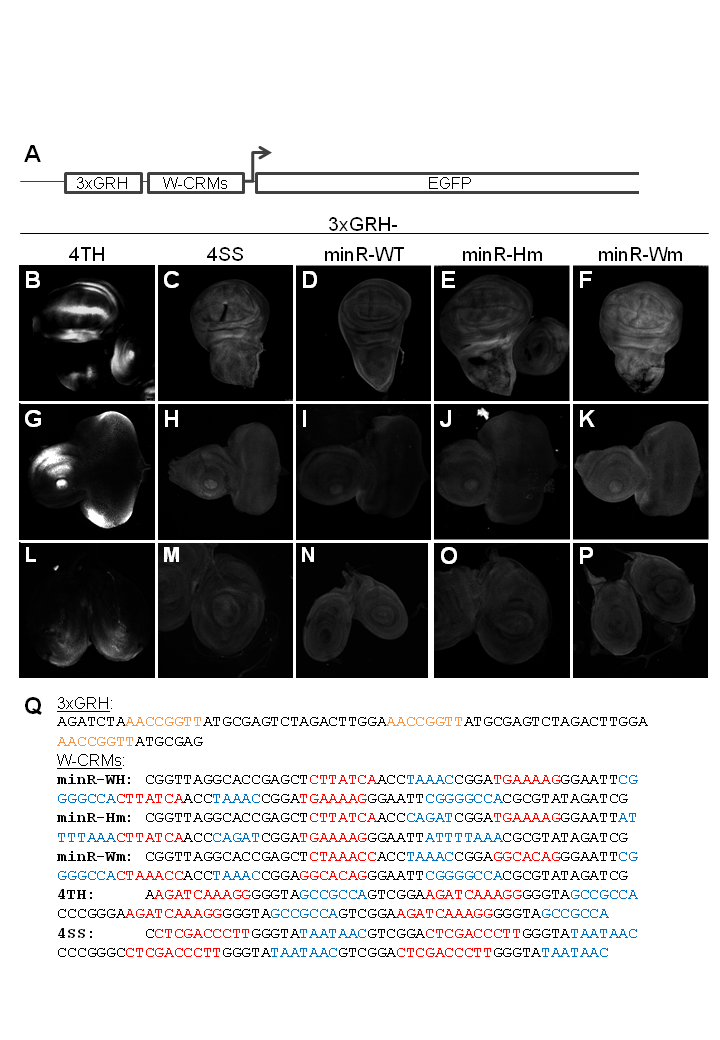

Supplement: Figure S12 — WGAWAW, r-Helper site pairs do not affect transcription in several other tissues outside the hematopoietic system. (A) A cartoon showing the structure of 3×GRH-W-CRM reporters, containing three Grainyhead (GRH) binding sites which provides basal activity in the tissues being tested and a W-CRM followed by a EGFP reporter gene. (B–P) Micrographs of wing (B–F), leg (G–K) and eye-antenna (L–P) discs from 3rd instar larvae carrying indicated 3×GRH-W-CRMs. 3×GRH-4TH contains four classic HMG-Helper site pairs, and displays high expression in regions where Wg is known to be expressed. 3×GRH-SS contains randon sequences and has the low level, ubiquitous pattern previously described [63]. 3×GRH-minR-WT along with the r-Helper (Hm) and WGAWAW (Wm) site mutant versions are all expressed in very similar patterns to 3×GRH-SS, with no hint of basal activation or Wg-dependent repression. (Q) Sequence information for the 3×GRH-W-CRM reporters. (TIF) [file pgen.1004509.s012.tif]

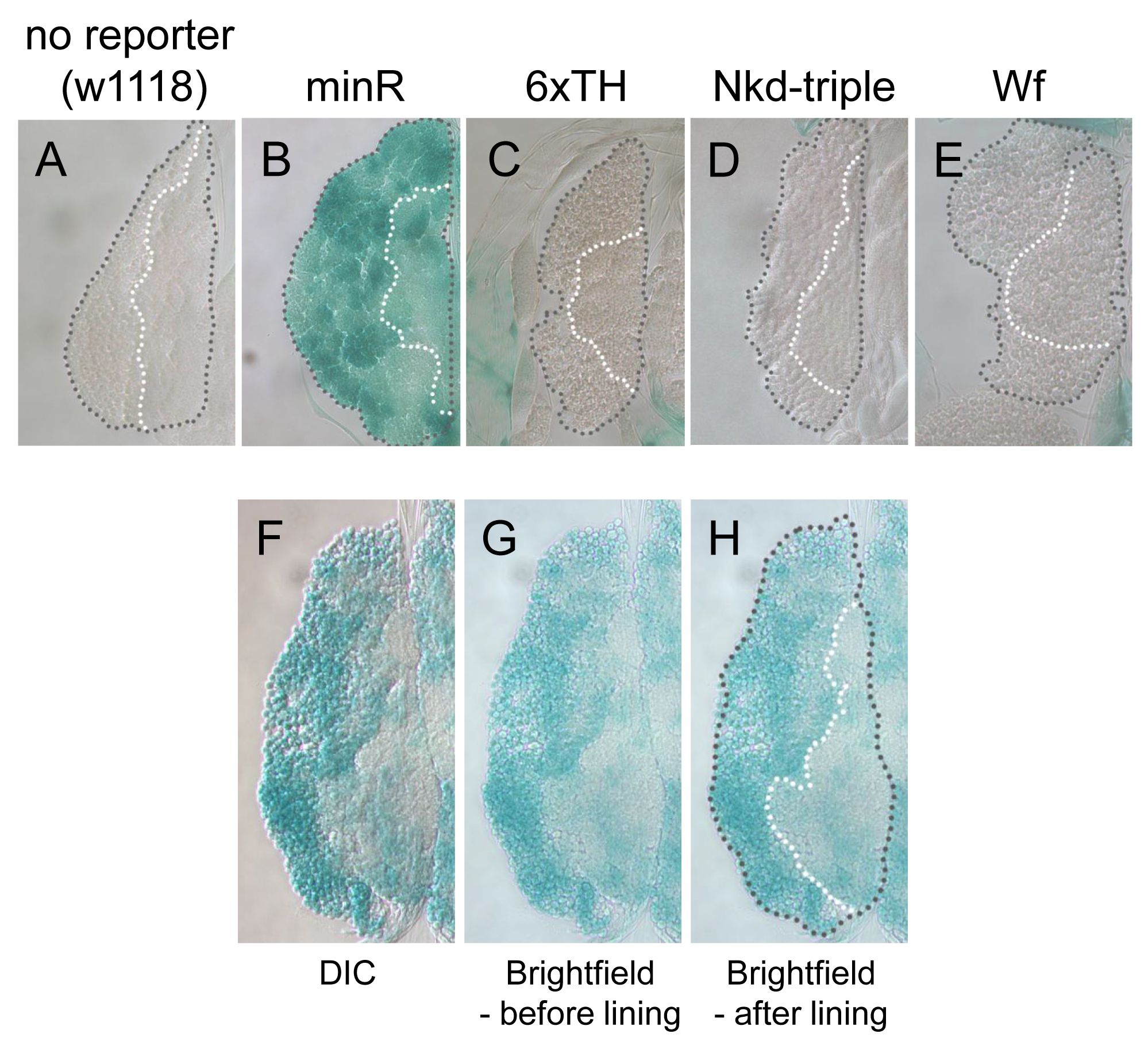

Supplement: Figure S13 — Several Wnt-activated W-CRMs have no detectable activity in the LG. (A–E) Comparison between minR and Wnt-activated reporters stained with X-gal. The minR reporter (B) shows strong staining in the CZ, while all the Wnt-activated W-CRMs tested (C–E), as well as the negative control w1118 (A), have no detectable staining. The minR reporter was stained for the same amoun of time as the other reporter lines, resulting in over-staining. (F) Micrograph of an older 3rd instar larval LG stained with X-gal, taken with DIC optics, highlighting the larger, less densely packed cells of the CZ. (G) Brightfield image of the same LG where the LacZ staining is more pronounced. (H) Brightfield image of the same LG where the DIC image was used to draw a broken white line separating the CZ and MZ. (TIF) [file pgen.1004509.s013.tif]
